# Dodder seedlings benefit from apoplastic nutrition by inducing SWEET15‐mediated unloading of sucrose from the host's phloem

**DOI:** 10.1111/nph.71270

**Published:** 2026-05-16

**Authors:** Maleen Hartenstein, Dominik Eydam, Antonia Klatt, Arne Greif, Peter Slaby, Ronja Burggraf, Franz Klebl, Isabell Albert, Uwe Sonnewald, Ruth Stadler, Markus Albert

**Affiliations:** ^1^ Department of Biology, Molecular Plant Physiology (MPP) Friedrich‐Alexander‐Universität Erlangen‐Nürnberg, FAU Erlangen‐Nürnberg Staudtstr. 5 91058 Erlangen Germany; ^2^ Department of Biology, Biochemistry Friedrich‐Alexander‐Universität Erlangen‐Nürnberg, FAU Erlangen‐Nürnberg Staudtstr. 5 91058 Erlangen Germany; ^3^ Department of Environmental Sciences, Organic Geochemistry University of Basel Bernoullistraße 30 4056 Basel Switzerland

**Keywords:** *Cuscuta*, parasitic plants, plant immunity, plant–plant interaction, sucrose transport proteins, SWEETs

## Abstract

- *Cuscuta* species are holoparasitic plants that penetrate host stems with haustoria, connect to the vasculature and exhaust water, nutrients and carbohydrates. Parasite seedlings possess only limited maternal reserves and the development of a vascular connection takes several days. Until then, an alternative access to host assimilates is critical for the survival of the parasite.
- Here, we investigated the sucrose flux and nutrition mechanisms of dodder during early infection. Using phloem‐mobile fluorescent tracers, we show that an apoplastic sucrose transfer occurs before symplastic connections between host and parasite are formed. We identified sucrose transporters of both dodder and host that are critical for assimilate transfers.
- CrSUT1, CrSUT3 and CrSWEET10 mediate sucrose transport during haustorium development. In the phloem of Arabidopsis, at the infection site, the sucrose‐exporter *AtSWEET15* is induced through basic leucine zipper transcription factors. CrSUT2 was identified as a transporter for sucrose uptake into the invading haustorium. The growth of dodder seedlings on *atsweet15* hosts is reduced, demonstrating the importance of the sucrose export by AtSWEET15 and availability within the apoplast.
- Our data highlight the relevance of apoplastic nutrition for the survival of parasitic plants. Dodder manipulates the host, causing it to release carbohydrates from its phloem during the initial infection.

## Graphical abstract

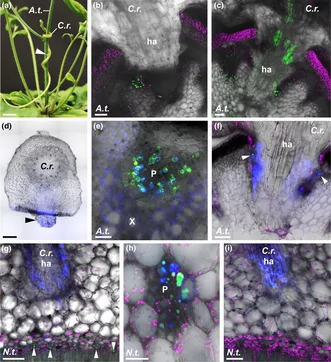

## Introduction

Nearly all land plants live a photoautotrophic lifestyle and do not depend on organic carbon sources since they can feed on the atmospheric carbon dioxide. However, *c*. 1% of the plants, more than 4700 species, live as parasitic plants, feeding on other plants as hemi‐ or holoparasites (Westwood *et al*., 2010; Nickrent, 2020). This often harms the host plants, and some parasitic plants cause significant agronomic damage to crops (Musselman *et al*., 2001; Parker, 2009; Runyon *et al*., 2010). While hemiparasites usually only extract water and solutes from their hosts, holoparasites also obtain fixed carbon in the form of sucrose. Many well‐known and agronomically important holoparasites include *Orobanche* spp., *Phelipanche* spp., both belonging to the Orobanchaceae (Westwood *et al*., 2010), as well as *Cuscuta* spp. from the Convolvulaceae (Vaughn, 2002; Albert *et al*., 2008).

The genus *Cuscuta* comprises of 150–200 species (McNeal *et al*., 2007), all of which are obligate holoparasitic plants without roots and leaves. *Cuscuta* spp. have a wide host range, and there are only a few host species that exhibit resistance to the parasite. For example, cultivated tomato (*Solanum lycopersicum*) shows pattern‐triggered immunity against *Cuscuta reflexa* (Hegenauer *et al*., 2016, 2017) after detecting a cell‐wall protein of the parasite (Hegenauer *et al*., 2020). To gain access to host plant assimilates, *Cuscuta* spp. twine around the host stem and develop haustoria to penetrate the host tissue and connect to its vasculature. The haustorium is a vital structure for all parasitic plants and the established connection between the parasite and host, including solute and carbon fluxes through the haustorium, is well‐documented (Jeschke *et al*., 1994; Hibberd & Jeschke, 2001). However, the nutrition strategies during the several days of haustorium development remain unclear. The haustorium formation is divided into three phases: the adhesive, the intrusive and the conductive phase (Yoshida *et al*., 2016). In the adhesive phase, *Cuscuta* spp. attach tightly to the host stem and epithelial cells facing the host stem elongate. Prehaustorial cells in the *Cuscuta* stem begin to proliferate and form a prehaustorial structure that points toward the host (Vaughn, 2002). During the intrusive phase, prehaustorial cells elongate rapidly and the haustorium breaks through the parasitic cortex to penetrate the host tissue (Lee, 2007). Searching hyphae, outgrowths of single haustorial cells, comparable to root hairs and pollen tubes, begin to grow into the surrounding tissue to locate the host vascular system (Dörr, 1969; Vaughn, 2003, 2006). The conductive phase starts after *c*. 1 wk postparasite onset, as soon as searching hyphae have reached host phloem and xylem and established a direct contact to the host vasculature (Vaughn, 2006; Yoshida *et al*., 2016; Shimizu & Aoki, 2019).

During the conductive phase, water, micronutrients, solutes (Jeschke *et al*., 1994; Hibberd & Jeschke, 2001), as well as carbohydrates, amino acids, phytohormones and systemic signals migrate from the host to the parasite (Jeschke *et al*., 1994; Birschwilks *et al*., 2006; Zhang *et al*., 2021, 2025). Furthermore, it was shown that even macromolecules are exchanged, since phloem‐mobile green fluorescent protein (GFP) (Haupt *et al*., 2001b), RNA (Roney *et al*., 2006; LeBlanc *et al*., 2012, 2013; Shahid *et al*., 2018) and viruses (Birschwilks *et al*., 2006) are being transferred from the host into *Cuscuta* spp. These findings demonstrate that symplastic connections are formed between *C. reflexa* and the host phloem via interspecific plasmodesmata. The time required to complete the infection and establish a symplastic connection depends on the host. In the parasitic interaction with *Arabidopsis thaliana* as a host for *C. reflexa*, the symplastic connection is formed at 6 d postinfestation (dpi). Haustorium development and penetration include meristem development, cell division and elongation and are thus energy‐ and time‐intensive processes. How *C. reflexa* survives the initial phases without starving and how the developing haustorial cells receive sufficient energy before the establishment of a vascular connection remains poorly understood. It has been shown that *Cuscuta campestris* seedlings can grow without a host on paper spindles soaked with glucose as an energy source (Bernal‐Galeano *et al*., 2022). Since *C. campestris* cannot establish a symplastic connection to this artificial host without a vascular system, this is an indication for apoplastic uptake of sugars from the paper spindles. In a natural host–parasite interaction, this would require sucrose export from the host phloem and an uptake into haustorial cells by sugar transport proteins. SUGARS WILL EVENTUALLY BE EXPORTED TRANSPORTERS (SWEETs) are passive, bidirectional sugar transporters that facilitate the transport of sucrose along a concentration gradient, typically functioning as cellular sugar exporters (Chen *et al*., 2012; Eom *et al*., 2015). By contrast, SUCROSE TRANSPORTERS (SUTs) are active proton symporters capable of transporting sucrose against a concentration gradient, making them typical sucrose importers for recipient cells (Riesmeier *et al*., 1992; Sauer & Stolz, 1994).

Here, we show how haustorial cells of *C. reflexa* are supplied with energy before the life‐saving vascular connections to the hosts are established. Since the disaccharide sucrose is the main form of long‐distance transport of carbon in plants (Lohaus, 2022), our studies focus on sucrose transport mechanisms mediated by SUTs and SWEETs in both the parasite and host, which are crucial for the cellular uptake and unloading of sucrose at the infection site. *Cuscuta reflexa* induces the expression of *AtSWEET15* in the host phloem and absorbs the released sucrose via CrSUT2 which is localized in the tip of the intruding haustorium. We used *atsweet15* knockout plants to investigate the importance of the sucrose export for the nutrition of the parasite. We show that esculin transfer from the phloem of *atsweet15* into the parasite is impaired and that dodder seedlings exhibit reduced growth.

## Materials and Methods

### Strains and growth conditions

*Arabidopsis thaliana* L. Columbia‐0 (Col‐0), *atsweet15* knockout (NASC ID: N68995; Chen *et al*., 2015) was cultivated under long‐day conditions (16 h : 8 h, light : dark) at 22°C and 60% humidity. *Arabidopsis thaliana* Col‐0 plants for protoplasts were cultivated under short‐day conditions (8 h : 16 h, light : dark). *Nicotiana tabacum* L. was cultivated in the glasshouse under long‐day conditions (16 h : 8 h, light : dark) at 22°C. *Cuscuta reflexa* Roxb. and *Cuscuta campestris* Yunck. were cultivated on *Coleus blumei* Benth. under long‐day conditions.

### Artificial induction of *C. reflexa* haustoria

The protocol for artificial induction of haustoria in *C. reflexa* was modified (Lachner *et al*., 2020): *C. reflexa* shoots were put between two Petri dishes, pressure applied, exposed to far‐red light for 1 h and incubated in darkness for 5 d. For the dye uptake into haustoria, *C. reflexa* shoots with artificially induced haustoria were incubated in 100 mM esculin or 0.3 mg ml^−1^ carboxyfluorescein‐diacetate (CFDA) or 0.3 mg ml^−1^ fluorescein for 2.5 h. The shoots were washed in Milli‐Q before the preparation for microscopy.

### Infection of host plants with *Cuscuta*

Infection of host plants with *C. reflexa* was performed as described (Hegenauer *et al*., 2017). For RNA sampling, *C. reflexa* stem pieces were coiled around a wooden stick and incubated for 24 h before infecting the host to promote time‐coordinated development of haustoria. As a negative control, *C. reflexa* shoots were collected without host contact or precoiling (−1 dpi). Tissue of *C. reflexa* on 0 dpi was harvested after 1 d of precoiling on a wooden stick. *Arabidopsis thaliana* samples on 0 dpi were collected right before infection with the precoiled *C. reflexa* stems. When taking samples for RNA extraction, the tissues of both species were separated from each other. Samples of at least three plants were pooled for each time point.

For the seedling growth assay, *C. campestris* seeds were scarified (Bernal‐Galeano *et al*., 2022) and germinated (Adhikari *et al*., 2025) following the published protocols. The seedlings were transferred to *A. thaliana* inflorescences 6 d postgermination.

### RNA isolation and qRT‐PCR

RNA from *A. thaliana* and *C. reflexa* was isolated according to protocol using the innuPREP Plant RNA Kit (Analytik Jena, Jena, Germany). RNA was transcribed into cDNA according to protocol using the QuantiTect Reverse Transcription Kit (Qiagen). The templates were amplified by PCR with the primers listed in Supporting Information Table S2. Primer pairs for reference genes amplified fragments of *CrActin* (GDKD01018661 (Olsen *et al*., 2016)) for *C. reflexa* and *AtUBQ10* (AT4G05320) for *A. thaliana* cDNA. For the quantitative reverse transcriptase polymerase chain reactions (qRT‐PCR), Brilliant III‐Fast SYBR® Green QPCR Master Mix (Agilent Technologies, Santa Clara, CA, USA) was used. The qRT‐PCR run was performed with the qTOWER iris Real‐time Thermocycler (Analytik Jena). The raw *C*_t_ values were used for ddCt analysis and the normalized expression was calculated using qPCRsoft 5.0 (Analytik Jena).

### Cloning

The sequences of the genes were retrieved from contigs of a *C. reflexa* Transcriptome Shotgun Assembly project (Olsen *et al*., 2016): CrSWEET10 (GDKD01017082), CrSUT1 (GDKD01024662), CrSUT2 (GDKD01000714/GDKD01018610) and CrSUT3 (GDKD01022886). To obtain the complete coding sequence of CrSUT1, a 5′‐RACE was performed with the SMARTer® RACE 5′/3′ Kit (Takara Bio, Kasatsu, Shiga, Japan) according to the manufacturer's instructions. For cloning, the coding sequences of *CrSWEET10*, *CrSUT1*, *CrSUT2* and *CrSUT3* were amplified via PCR from *C. reflexa* cDNA with primer pairs (Table S2) that introduced *BsaI* sites and *pENTR‐BsaI*‐specific overhangs on the 5′ and 3′ end of the PCR products. Ligation of the PCR fragments into *pENTR‐BsaI* (Binder *et al*., 2014) was performed via Golden Gate Cloning (Engler *et al*., 2008). The inserts in *pENTR‐BsaI* were recombined into the yeast expression vector *pAG426GDP* (Addgene plasmid #14156; http://n2t.net/addgene:14156; RRID:Addgene_14156; Alberti *et al*., 2007) and the plant expression vectors *pMDC43* or *pMDC83* (Curtis & Grossniklaus, 2003) with Gateway LR reactions, respectively. The following constructs were created: *pAG426GPD‐CrSWEET10*, *pMDC43‐CrSWEET10‐EGFP*, *pAG426GPD‐CrSUT1*, *pMDC83‐CrSUT1‐EGFP*, *pAG426GPD‐CrSUT2*, *pMDC83‐CrSUT2‐EGFP*, *pAG426GPD‐CrSUT3*, and *pMDC43‐EGFP‐CrSUT3*.

For the *pAtSWEET15::GUS* reporter lines, a 2000‐bp promoter region of *AtSWEET15* (AT5G13170) was amplified from *A. thaliana* gDNA with primer pairs (Table S2) that introduced *BsaI* sites and *pENTR‐BsaI*‐specific overhangs on the 5′ and 3′ end of the PCR product. Ligation of the PCR fragment into *pENTR‐BsaI* was performed as described above. Subsequently, the promoter insert was cloned into *pBASTA‐GUS* (Rottmann *et al*., 2016) by LR reaction, yielding *pBASTA‐pAtSWEET15::GUS* for stable transformation in *A. thaliana*.

### Functional characterization of sucrose transport properties by heterologous expression in baker's yeast

*pAG426GDP‐CrSUT1*, *pAG426GDP‐CrSUT3*, *pAG426GPD* (Addgene plasmid #14156; http://n2t.net/addgene:14156; RRID:Addgene_14 156; Alberti *et al*., 2007) and *NEV‐UmSRT1* (Wippel, 2011) were transformed into *Saccharomyces cerevisiae* SEY2101 (*MAT*a, *ura3‐52*, *leu2*,*3–112*, *ade2‐1* and *suc2‐Δ9*) via lithium acetate mediated transformation (Soni *et al*., 1993). Sucrose uptake measurements and kinetics of transformed yeast were performed as described with 100 mM ^14^C‐labeled sucrose (Sauer & Stadler, 1993; Wahl *et al*., 2010).

### Protoplast transformation and loading of fluorescent dyes

Leaf mesophyll protoplasts from Col‐0 plants were prepared as described (Drechsel *et al*., 2011). Protoplast transformation with *pMDC43‐CrSWEET10‐EGFP*, *pMDC83‐CrSUT1‐EGFP*, *pMDC83‐CrSUT2‐EGFP* or *pMDC43‐EGFP‐CrSUT3*, respectively, followed by incubation in 1 mM esculin, was carried out according to the method described by Rottmann *et al*. (2018).

To load fluorescent dyes (esculin or CFDA) into the host plants and observe dye content in the attached *C. reflexa* haustoria, the source leaves of the host plants were roughened using sandpaper, and 100 mM esculin or 0.3 mg ml^−1^ CFDA was applied. The treated leaves were covered with parafilm, and microscopic observation at the infection site was conducted 3 h after loading (Mehdi *et al*., 2019).

### Tissue embedding and *in situ* mRNA hybridization

GUS‐stained tissue samples were destained in 70% EtOH and embedded in Technovit® 7100 (Kulzer GmbH) according to the manufacturer's protocol and cut with a Leica Ultracut R Ultramicrotome (Leica Microsystems, Wetzlar, Germany) in 5 μm thin sections.

For the *in situ* hybridization, pieces of *C. reflexa* stems and haustoria were fixed in FAA (50% Ethanol (v/v), 5% Glacial Acetic Acid (v/v), 3.7% Formaldehyde (v/v)) and dehydrated through an ethanol series. The samples were embedded in methacrylate (Stadler & Sauer, 1996), and sections (3 μm) were prepared using a Leica Ultracut R Ultramicrotome (Leica Microsystems).

For the probe synthesis, fragments (200–300 bp) of the coding sequences of the sucrose transporters were amplified with the primers listed in Table S2. The probe synthesis and *in situ* hybridization were conducted after the protocol (Gramma & Wahl, 2023) with the following exceptions: The probes were not fragmented and TNM‐50 instead of PVA‐TNM‐50 buffer was used. The antisense probes for *CrSUT3* and *CrSWEET10* were pooled.

### Microscopy

For the detection of GFP, carboxyfluorescein (CF) and esculin fluorescence, images were taken on a Leica TCS SP5 II confocal laser scanning microscope (Leica Microsystems). The 488‐nm argon laser was used for excitation of GFP, CF and Chl autofluorescence. The 405‐nm diode was used for excitation of esculin fluorescence. Detection windows ranged from 507 to 531 nm for GFP and CF, 630 to 728 nm for Chl autofluorescence and 440 to 465 nm for esculin. Images of GFP together with esculin were taken in a sequential scanning mode. Images of GUS‐stained plants were taken with a ZEISS Axio Zoom.V16 (Carl Zeiss Microscopy, Oberkochen, Germany). Image processing was performed with Leica Las Af (Leica Microsystems), Zeiss Zen pro (Carl Zeiss Microscopy) and Pixelmator Pro (Pixelmator Team Ltd).

## Results

### Penetrating haustoria take up sucrose from the apoplastic space

**Fig. 1:**
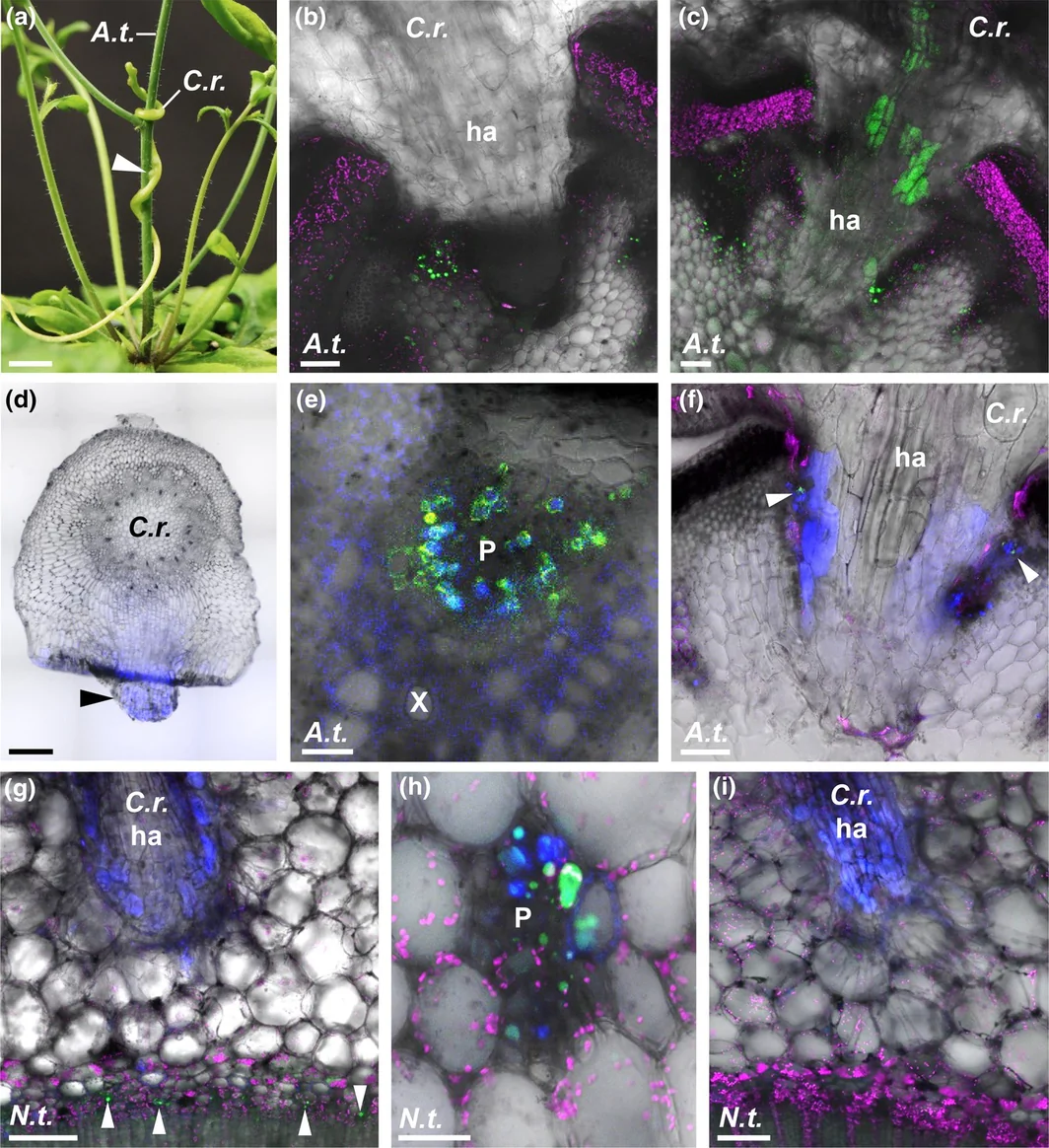
*Cuscuta reflexa* infection sites. (a) *C. reflexa* (*C. r*.) winding around its host plant *Arabidopsis thaliana* (*A. t*.). White arrowhead points at the swollen tissue of the *C. reflexa* stem at the infection site, from which a haustorium emerges. (b, c) Cross‐section of the infection site of *C. reflexa* infecting *A. thaliana*, 5 d postinfestation (dpi) (b) and 6 dpi (c). *A. thaliana* leaves were loaded with carboxyfluorescein diacetate (CFDA) to monitor the specific timing of the establishment of a symplastic connection between the haustorium and the host phloem. 5 dpi, no transfer of carboxyfluorescein (CF, green) is visible. 6 dpi, transfer of CF is visible. (d) Section of an artificially induced haustorium of *C. reflexa* without host contact. Before sectioning, stems with haustoria were incubated in solution containing membrane impermeable esculin (blue) for 2.5 h. (e, f) *A. thaliana* infected with *C. reflexa* 4 dpi. *A. thaliana* source leaves were loaded with esculin and CFDA. (e) Close‐up view of *A. thaliana* phloem (P) distant to the infection site showing esculin fluorescence in blue and CF fluorescence in green. X (xylem). (f) Haustorium intrusion site of *A. thaliana* infected with *C. reflexa* 4 dpi. Arrowheads indicate *A. thaliana* phloem adjacent to the haustorium (ha), which has absorbed esculin but not CF. (g) *pAtSUC2::GFP* in *Nicotiana tabacum* (*N. t*.) infected with *C. reflexa* 6 dpi. *N. tabacum* source leaves were loaded with esculin. Sections of the infection site were prepared 3.5 h after loading with esculin. Arrowheads indicate GFP fluorescence in host phloem. (h, i) *N. tabacum* (*N. t*.) infected with *C. reflexa* 6 dpi. *N. tabacum* source leaves were loaded with esculin and CFDA. (h) Close‐up view of *N. tabacum* phloem (P) distant to the infection site showing esculin fluorescence in blue and CF fluorescence in green. (i) Section of the infection site of the same plant as in (h) shows haustorium intrusion site of *N. tabacum* (*N. t*.) infected with *C. reflexa* 6 dpi. The representative confocal images show fluorescence of CF and GFP in green, esculin in blue, autofluorescence of chloroplasts and lignin in magenta. Bars: (a) 5 mm; (b, c, f) 100 μm; (e) 25 μm; (d) 500 μm; (g, i) 2500 μm; (h) 50 μm.

To study *C. reflexa* nutrition before the establishment of a symplastic phloem–phloem connection between host and parasite, we analyzed the timing of the transition from the intrusive to the conductive infection phase in the interaction between *C. reflexa* and *A. thaliana* (Fig. 1a). To investigate symplastic connections, the membrane‐permeable compound carboxyfluorescein diacetate (CFDA) can be applied to the leaves of the host plant tissue. Within the leaf cells, CFDA is converted by cytosolic esterases into the membrane‐impermeable, fluorescent compound CF. While CF in mesophyll cells cannot enter the phloem vessels, CF in sieve element–companion cell complexes has access to phloem transport and can be used as a marker for symplastic phloem unloading. The earliest time point of CF transfer could be observed 6 d after parasite onset, whereas no CF was detectable in *C. reflexa* tissue at 5 dpi (Fig. 1b,c).

To investigate whether a functional sucrose transporter is present and potentially facilitates cellular sucrose uptake in early developing haustoria of *C. reflexa*, we used the fluorescent sucrose analog esculin, which is recognized by sucrose transporters (Gora *et al*., 2012). Haustoria were artificially induced in isolated *C. reflexa* shoots and incubated in a solution containing esculin. Sections of the developing haustorium revealed that esculin was taken up by cells of artificially induced haustoria (Fig. 1d). To ensure that the fluorescent dye was not taken up by vesicle‐mediated processes, we incubated artificially induced haustoria with membrane‐impermeable fluorescein and, as a control, with membrane‐permeable CFDA. While incubation with CFDA resulted in fluorescence labeling within the cells of the haustorium, fluorescein was unable to enter the cells (Fig. S1A,B). These results show that the haustorial cells are not able to take up fluorescent dyes via endocytosis. Together with the observed uptake of esculin, the data suggest the existence of a functional sucrose transporter in the cells of the artificially induced haustorium.

*Arabidopsis thaliana* was infected with *C. reflexa* and loaded with esculin and CFDA at 4 dpi (Fig. 1e,f). Fig. 1(e) shows the localization of esculin and CF in the *A. thaliana* phloem of the stem close to the infection site. The section of the haustorium intrusion site shows that esculin is transferred from the *A. thaliana* phloem into haustorial cells, whereas CF is not transferred (Fig. 1f). This indicates that a symplastic connection between haustorium and host phloem is not yet established and that esculin is transferred apoplastically.

Esculin uptake was then analyzed in the host–parasite system of *N. tabacum* and *C. reflexa* (Fig. 1g–i). In *A. thaliana*, *C. reflexa* haustoria grow rather quickly through the thin cortex and it is difficult to distinguish between the intrusive and conductive phases. In *N. tabacum*, however, the onset of the inductive phase occurs later compared with *A. thaliana* due to its thick cortex. Within 6 dpi, the haustorium of *C. reflexa* is still distinctly in the intrusive phase and has not yet reached the vasculature of *N. tabacum* (Figs 1g,i, S1C). This advantageous shoot anatomy allows a more convenient time frame to monitor the haustorium intrusion.

To ensure that imaging is performed before a symplastic connection is established, we used *N. tabacum* expressing *pAtSUC2::GFP* (Imlau *et al*., 1999) for infection with *C. reflexa* (Figs 1g, S1C). The *AtSUC2*‐promoter is exclusively active in companion cells, allowing for the detection of phloem‐mobile GFP fluorescence throughout the phloem. Six dpi, esculin was applied to *N. tabacum* source leaves and, like sucrose, is loaded into source leaf phloem via the sucrose carrier SUT1/SUC2 (Sivitz *et al*., 2007) and transported to sink tissues. Esculin could be detected in attached *C. reflexa* haustoria 3.5 h after loading *N. tabacum* (Figs 1g, S1C). There is no sign for a symplastic connection to the phloem because the searching hyphae of *C. reflexa* are still three to five cell layers distant from the phloem of *N. tabacum* and phloem‐mobile GFP from *pAtSUC2::GFP* in *N. tabacum* is not yet detectable in the haustorium or the parasitic vasculature (Fig. S1C). As presented in previous work, phloem‐mobile GFP is detectable in high amounts in *Cuscuta* at 14–16 dpi after the establishment of a symplastic connection (Fig. S1D; Haupt *et al*., 2001b). Additionally, *N. tabacum* was infected with *C. reflexa* and loaded with esculin and CFDA at 6 dpi (Fig. 1h,i). Fig. 1(h) shows the localization of esculin and CF in the *N. tabacum* phloem. A section of the haustorium intrusion site shows that esculin is transferred from the *N. tabacum* phloem into haustorial cells, whereas CF is not transferred (Fig. 1i). Fig. S1(E) shows the transfer of CF from *N. tabacum* to a *C. reflexa* haustorium at 8 dpi, when the symplastic connection is established.

Taken together, the data demonstrate the release of the sucrose analog esculin from phloem cells of the stems of *N. tabacum* and of *A. thaliana* into the apoplast, as well as the uptake of esculin by *C. reflexa* haustorial cells from the apoplastic space during the intrusive phase. Apoplastically available sucrose would require the induction of local phloem unloading in the host at the infection site. Therefore, we postulate not only a sucrose importer in haustorial cells of *Cuscuta*, but also a sucrose exporter in the phloem of the host.

### Expression of sucrose transporter genes during the infection

Since the uptake of esculin from the apoplast into young *C. reflexa* haustoria is an indication for an active sucrose transporter in this tissue, we searched for genes with homology to sucrose transporters that are expressed during the infection. Initially, we scanned RNA‐seq data of infection sites between *C. japonica* and *Glycine max* for sucrose transporter candidate transcripts upregulated at different time points (Ikeue *et al*., 2015). Then, we screened for homologs in data provided by the *C. reflexa* Transcriptome Shotgun Assembly project (Olsen *et al*., 2016). We identified three *SUT* genes, *CrSUT1* to *3* and one *SWEET* gene in *C. reflexa*. The sequences are listed in the Table S1. We named the *SWEET* gene *CrSWEET10* based on the highest blast homology to *AtSWEET10* of *A. thaliana* (Altschul *et al*., 1990; UniProt Consortium, 2023). Additionally, CrSWEET10 clustered with AtSWEET10 and *Vitis vinifera* SWEET10 (VvSWEET10) in a phylogenetic analysis (Fig. S2), which was conducted with Mega11 (Tamura *et al*., 2021). CrSUT2 clustered with type IIA SUT2/SUC3 proteins. CrSUT3 and CrSUT1 clustered together in the group of type I SUTs (Fig. S2).

**Fig. 2:**
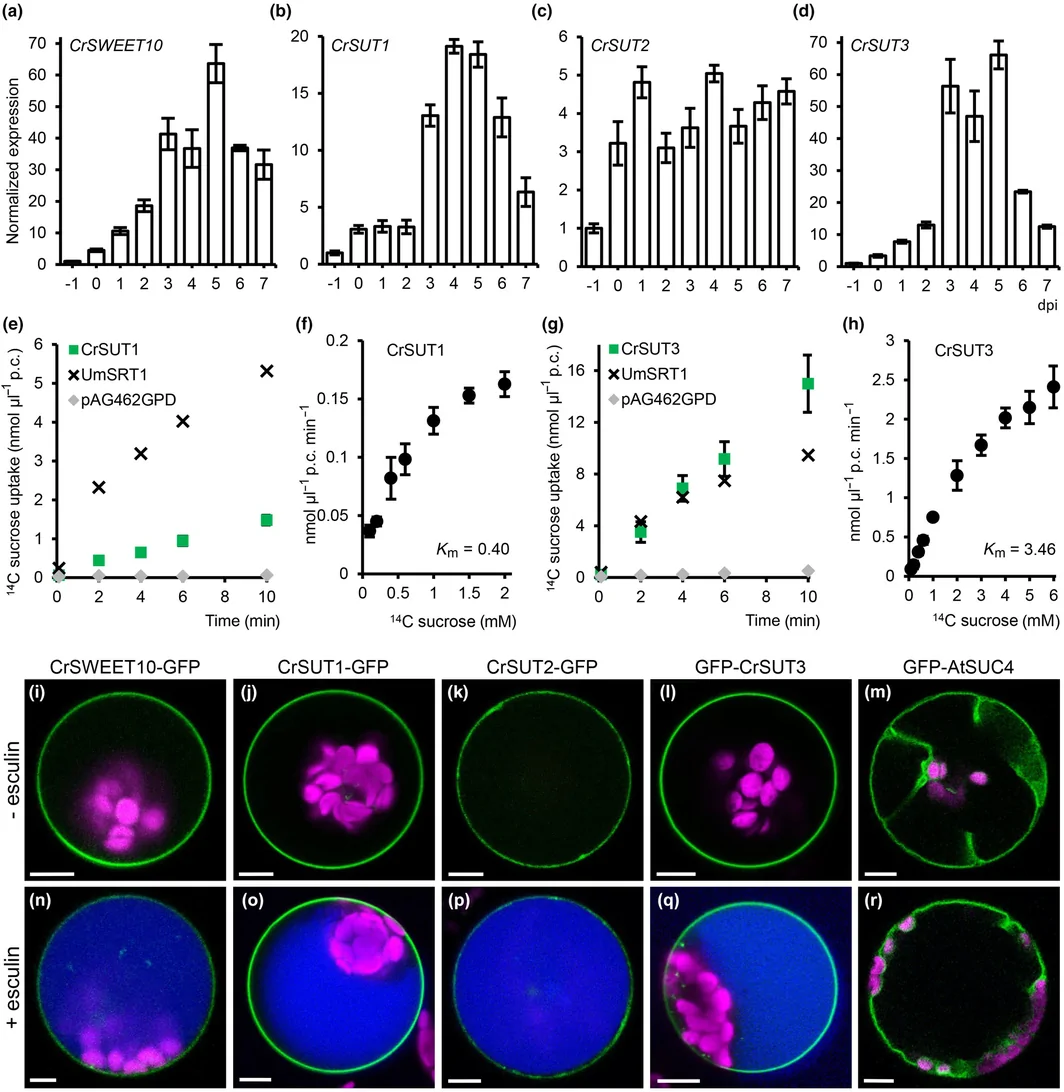
Expression analyses and sucrose transport properties of *CrSWEET10*, *CrSUT1*, *CrSUT2* and *CrSUT3*. (a–d) Representative normalized expression of *CrSWEET10* (a), *CrSUT1* (b), *CrSUT2* (c) and *CrSUT3* (d) during infection of *Arabidopsis thaliana* by *Cuscuta reflexa*, measured by quantitative reverse transcriptase polymerase chain reactions (qRT‐PCRs). Untreated *C. reflexa* shoot was harvested on −1 d postinfestation (dpi) and served as the calibrator for ddCt calculations. Precoiled *C. reflexa* shoots were harvested on 0 dpi. Samples for each time point were pooled from at least three independent biological replicates. *CrActin* (GDKD01018661; Olsen *et al*., 2016) was used as the reference gene. The data represent the means of three technical replicates ± SD. (e–h) ^14^C sucrose uptake of transgenic *Saccharomyces cerevisiae* SEY2101 transformed with the yeast expression vector pAG426GPD with the cDNA inserts of *CrSUT1* (e, f) and *CrSUT3* (g, h). Measurements were obtained from three independent transformants. *Ustilago maydis SRT1* (black) served as the positive and the empty vector (gray) as the negative control. (e, g) ^14^C sucrose uptake of transgenic *S. cerevisiae* SEY2101 over time. (f, h) Michaelis–Menten kinetics of CrSUT1 (f) and CrSUT3 (h): reaction velocity [nmol μl^−1^ p.c. min^−1^] vs ^14^C sucrose concentration [nM]. The *K*_m_ value [mM] was calculated from the reciprocal transformation of the Michaelis–Menten kinetics to a Lineweaver–Burk function (Lineweaver & Burk, 1934). p.c., packed cells. (i–p) Subcellular localization and esculin uptake analysis by heterologous expression of *CrSWEET10‐GFP*, *CrSUT1‐GFP*, *CrSUT2‐GFP* and *GFP‐CrSUT3* in *A. thaliana* mesophyll protoplasts. (i–l) Subcellular localization of CrSWEET10‐GFP (e), CrSUT1‐GFP (f), CrSUT2‐GFP (g) and GFP‐CrSUT3 (h) in the plasma membrane of *A. thaliana* mesophyll protoplasts. (m) GFP‐AtSUC4 localizes to the tonoplast and served as the negative control for esculin uptake. (n–r) Transformed protoplasts after incubation with 1 mM esculin for 30 min. GFP fluorescence is shown in green, autofluorescence of chloroplasts is shown in magenta, and esculin fluorescence is shown in blue. Bars, 10 μm.

To verify the expression of the putative *C. reflexa* transporter genes during the host–parasite interaction, qRT‐PCRs were performed (Fig. 2a–d). For this, *A. thaliana* was infected with *C. reflexa*, and samples of the infection site of *C. reflexa* were collected at different time points for cDNA preparation.

*CrSWEET10* and *CrSUT3* showed similar expression time courses. Transcripts of both genes started to accumulate in the adhesive phase and peaked at more than 60‐fold in the intrusive phase at 5 dpi (Fig. 2a,d). Transcripts levels of *CrSUT1* remained low during the adhesive phase (0–2 dpi), increased rapidly during the intrusive phase after 3 dpi and reached their maximum at 4 dpi with a 19‐fold increase (Fig. 2b). The expression of *CrSUT2* was also elevated compared with the uninfected *C. reflexa* sample, although not as pronounced as the above‐mentioned genes, increasing by up to fivefold (Fig. 2c). The data indicate that all four sucrose transporters are expressed during the infection process and were therefore further investigated.

### Sucrose transport properties of *C. reflexa* sucrose transporters

The identified *C. reflexa* sucrose transporter candidates were functionally characterized for their quantitative sucrose transport capacities by heterologous expression in *Saccharomyces cerevisiae*. The coding sequences of the respective genes were cloned as cDNA in the yeast expression vector pAG426GPD and transformed in the *S. cerevisiae* strain SEY2101. Correct targeting in the yeast plasma membrane was confirmed by confocal microscopy of the respective GFP‐fusion proteins. CrSUT2 did not localize correctly in the yeast plasma membrane, making it impossible to obtain reliable sucrose uptake measurements. CrSWEET10 was not tested in this assay since SWEETs function as passive bidirectional transporters, making the accumulation of ^14^C sucrose within the yeast cells not measurable.

*Saccharomyces cerevisiae* expressing *CrSUT1* and *CrSUT3* were supplied with ^14^C sucrose and cellular sucrose uptake was measured dependent on the substrate concentration. Transformation with *CrSUT1* enabled *S. cerevisiae* to take up ^14^C sucrose (Fig. 2e). Michaelis–Menten kinetics revealed that the *K*_m_ value of CrSUT1 equals 0.40 mM and the maximum uptake rate of ^14^C sucrose was at *V*_max_ = 0.18 nmol μl^−1^ p.c. min^−1^ (Fig. 2f). Yeast cells expressing *CrSUT3* were able to accumulate ^14^C sucrose (Fig. 2g). The *K*_m_ value of CrSUT3 equals 3.46 mM, and the maximum uptake rate of ^14^C sucrose was at *V*_max_ = 3.08 nmol μl^−1^ p.c. min^−1^ (Fig. 2h). All measurements were carried out at pH 5.5 as it matches the natural extracellular pH of common plant cells. For the ^14^C sucrose uptake measurements, UmSRT1 served as the positive and the empty vector *pAG426GPD* as the negative control (Fig. 2e,g).

A second method was applied to determine whether all CrSUTs and CrSWEET10 function as sucrose transporters. A protoplast esculin assay was performed, which was specifically developed to analyze sucrose transport activities for proteins that could not be analyzed in *S. cerevisiae* (Rottmann *et al*., 2018). *Arabidopsis thaliana* mesophyll protoplasts were transiently transformed with *GFP*‐fusion constructs of the respective sucrose transporters and the uptake of the fluorescent sucrose analog esculin was observed. CrSWEET10‐GFP, CrSUT1‐GFP, CrSUT2‐GFP and GFP‐CrSUT3 were all targeted to the plasma membrane of the protoplasts (Fig. 2i–l). For CrSUT2‐GFP, GFP fluorescence was additionally visible in the endoplasmic reticulum, which indicates that parts of the fusion proteins were not fully sorted to the plasma membrane (Fig. 2k). After incubation with 1 mM esculin for 40 min, protoplasts expressing *CrSWEET10‐GFP*, *CrSUT1‐GFP*, *CrSUT2‐GFP* and *GFP‐CrSUT3* had taken up esculin (Fig. 2n–q). GFP‐AtSUC4 was used as the negative control since it localizes to the tonoplast (Fig. 2m) and therefore does not enable the protoplasts to accumulate esculin (Fig. 2r). These results demonstrate that all observed *C. reflexa* proteins can be classified as functional sucrose transporters that localize to the plasma membrane. Although sequence analysis and comparisons with SWEET homologs from other species indicate that CrSWEET10 functions primarily as a sucrose transporter, transport of glucose or fructose cannot be ruled out.

### *In situ* localization of *CrSWEET10* , *CrSUT1* , *CrSUT2* and *CrSUT3* mRNA

**Fig. 3:**
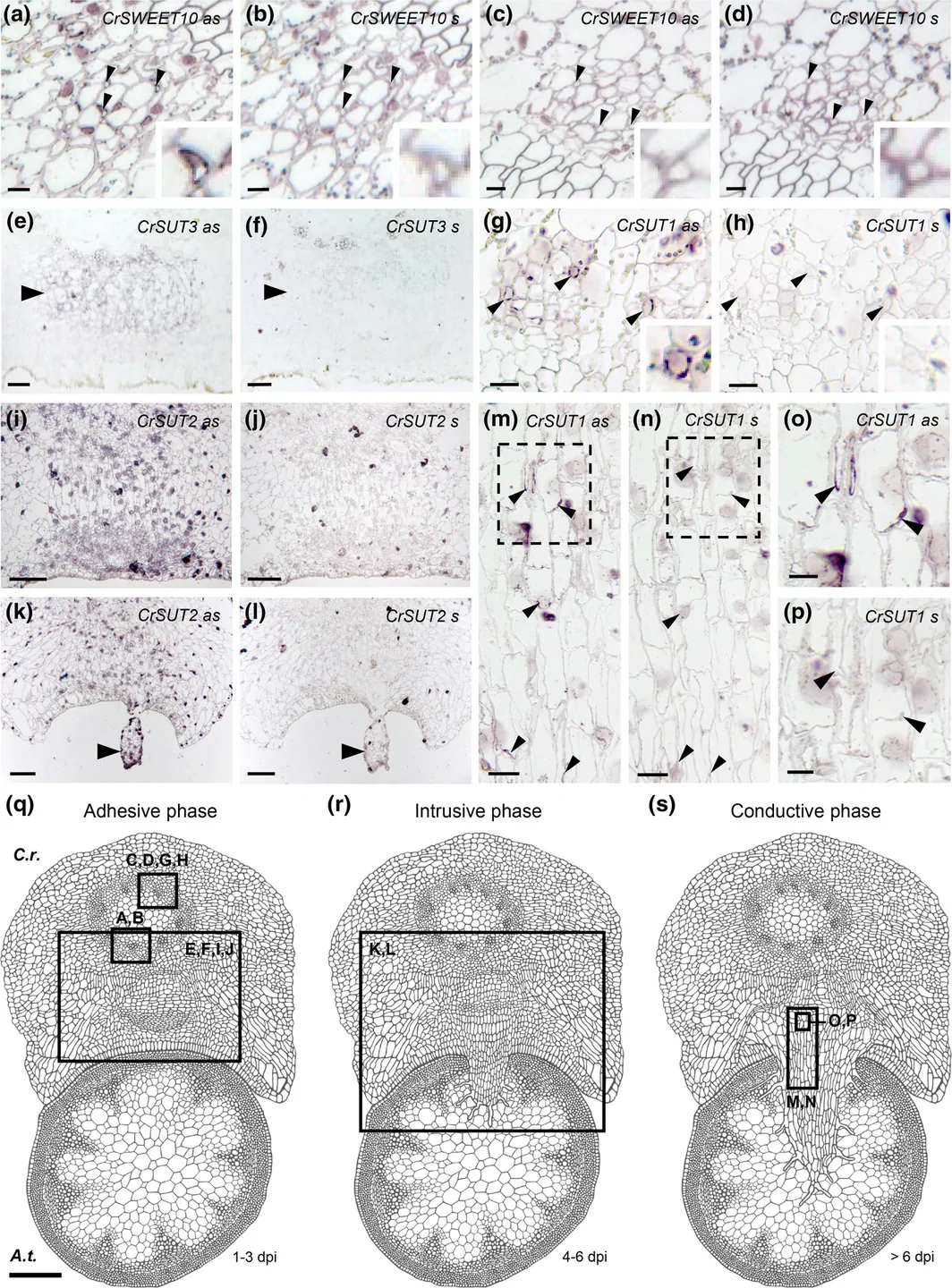
*In situ* hybridization of *CrSWEET10*, *CrSUT1*, *CrSUT2* and *CrSUT3* mRNA. (a–j) Adhesive phase, (k–l) intrusive phase (m–p) and conductive phase. (a–d) Cross‐sections of *Cuscuta reflexa* stem in the adhesive phase, hybridized with *CrSWEET10* probes. *CrSWEET10* antisense (a) and sense (b) probes in phloem bundle facing the infection site. Same section as in (a) and (b) shows incubation with *CrSWEET10* antisense (c) and sense (d) probes in phloem bundle distant to the infection site. Arrowheads point at companion cells. Boxes show the close‐up view of one representative companion cell. Staining is only visible in companion cells of (a). (e, f) Sections of *C. reflexa* stem during the adhesive phase, hybridized with *CrSUT3* antisense (e) and sense (f) probes. Arrowheads point at prehaustorial tissue. (g, h) Sections of phloem bundles in *C. reflexa* stem during the adhesive phase, incubated with *CrSUT1* antisense (g) and sense (h) probes. Arrowheads point at companion cells. Boxes show the close‐up view of one representative companion cell. (i, j) Sections of *C. reflexa* stem during the adhesive phase, hybridized with *CrSUT2* antisense (i) and sense (j) probes. (k, l) Sections of *C. reflexa* stem during the intrusive phase, hybridized with *CrSUT2* antisense (k) and sense (l) probes. Arrowheads point at the intruding haustorium. (m–p) Longitudinal sections of the *C. reflexa* haustorium in the conductive phase, hybridized with *CrSUT1* antisense (m, o) and sense (n, p) probes. (o, p) Enlarged images in (m, n) as indicated through the boxes. Arrowheads point at phloem cells in the haustorium. (q–s) Schematic overview of the exact locations of all image sections across the different phases. Bars: (a–d) 25 μm; (e, f) 200 μm; (g, h) 25 μm; (i–l) 100 μm; (m, n) 50 μm; (o, p) 25 μm. *as*, antisense; *s*, sense. Bar, 250 μm.

To investigate the spatiotemporal expression patterns of *CrSWEET10*, *CrSUT1*, *CrSUT2* and *CrSUT3* in *C. reflexa* during the infection, we performed *in situ* hybridizations of the respective mRNAs. Sections of methacrylate‐embedded *C. reflexa* stems and haustoria in the adhesive (1–3 dpi), intrusive phase (4–6 dpi) and conductive phase (> 6 dpi) were incubated with antisense or sense probes for the respective mRNAs (Fig. 3). The time points were selected to detect the maximum expression of the respective genes based on the results of the qRT‐PCR analyses. For *CrSWEET10*, an mRNA‐specific signal was detected in companion cells of the *C. reflexa* stem facing the infection site during the adhesive phase (Fig. 3a,b). In the same tissue section, companion cells in vascular bundles at the opposite side of infection did not show staining (Fig. 3c,d). No signal was visible in companion cells of any vascular bundle in the *C. reflexa* stem after incubation with the *CrSWEET10* sense probe, which served as the negative control (Fig. 3b,d). After incubation of sections with the *CrSUT3* antisense probe, staining was detected specifically in basal prehaustorial cells during the adhesive phase (Fig. 3e,f). The staining remained visible in the same tissue in the intrusive phase (Fig. S3A,B). Signals of *CrSUT1* antisense probes were visible in companion cells within vascular bundles of the *C. reflexa* stem (Fig. 3g,h) and in developing phloem cells of haustoria (Fig. 3m–p). Staining of the *CrSUT2* antisense probe was detectable at the tip of developing haustoria during the adhesive phase (Fig. 3i,j) and in haustoria of the intrusive phase (Fig. 3k,l). However, there was a high background staining of the *CrSUT2* antisense probe. Additionally, *CrSUT*2‐specific staining was detected in apical meristem of the *C. reflexa* shoot (Fig. S3C,D). The respective sense probes of *CrSUT3*, *CrSUT1* and *CrSUT2* did not show any staining (Figs 3f,h,j,l,m,p, S3B,D). In summary, the mRNA levels of *CrSWEET10* and *CrSUT1‐3* were abundant at the same time points, as detected by the qRT‐PCR experiments.

### *AtSWEET15* expression is induced by *C. reflexa* at the infection site

**Fig. 4:**
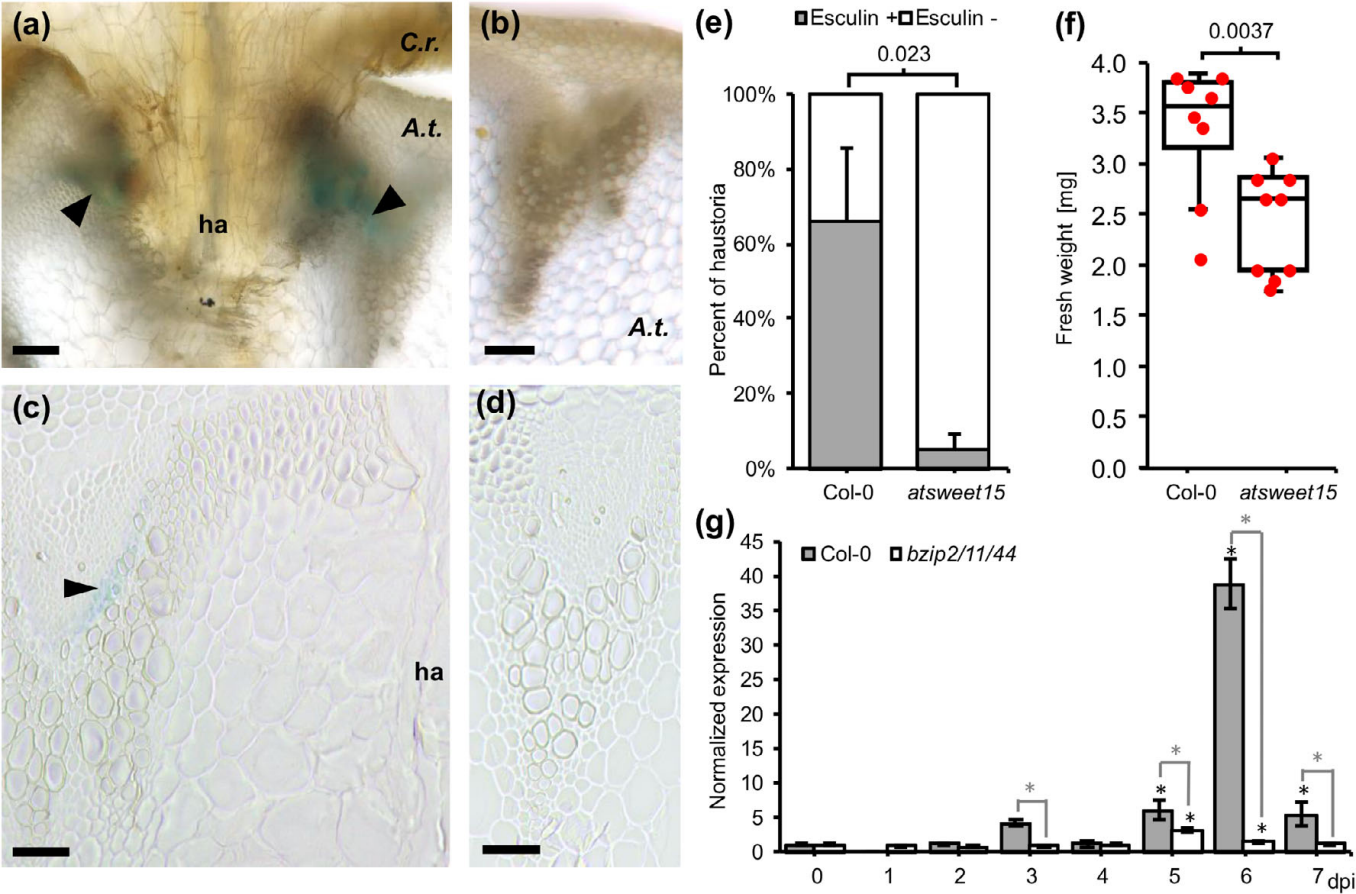
Functional analyses of *AtSWEET15* in *Arabidopsis thaliana* (*A.t*.) invaded by *Cuscuta reflexa* (*C. r*.) haustoria. (a, b) β‐glucuronidase (GUS)‐stained section of *A. thaliana* Col‐0 *pAtSWEET15::'GUS*, infected with *C. reflexa* for 8 d. (a) GUS staining (arrowheads) is visible in the phloem adjacent to the intruding haustorium (ha). (b) Vascular bundle of *A. thaliana* of the same section as in (a), distant to the infection site, shows no GUS staining. (c, d) Thin sections of GUS‐stained plants as in (a, b) embedded in Technovit® 7100. (c) Vascular bundle of *A. thaliana* adjacent to the *C. reflexa* haustorium (ha) shows blue staining (arrowhead) in phloem cells. (d) A vascular bundle of the same section distant to the infection site shows no staining. (e) Host plants were loaded with esculin and the graphs show the percentage of *C. reflexa* haustoria with (+) or without (−) detection of esculin fluorescence. Esculin was transferred from host plants *A. thaliana* Col‐0 (*n* = 35 haustoria on 9 host plants) and *atsweet15* (*n* = 30 haustoria on 11 host plants) to *C. reflexa* haustoria, 4 dpi. (f) Growth assay of *Cuscuta campestris* seedlings on *A. thaliana* Col‐0 (*n* = 8) and *atsweet15* (*n* = 9). The box plots show the median of the fresh weight of the seedlings at 4 dpi. Red dots show independent values. The *P*‐values for (e, f) were calculated using a one‐tailed Welch's *t*‐test and are displayed above the graphs. (g) Representative normalized expression of *AtSWEET15* during infection of *A. thaliana* Col‐0 or *bzip2/11/44* by *C. reflexa*, measured by quantitative reverse transcriptase polymerase chain reactions (qRT‐PCRs). Samples for each time point were pooled from at least three independent biological replicates. *AtUBQ10* (AT4G05320) was used as the reference gene. The data represent the means of three technical replicates ± SD. Black asterisks mark time points that differ significantly from day 0 within a genotype (Col‐0 or *bzip2/11/44*; one‐way ANOVA). Gray asterisks mark significant differences between Col‐0 and *bzip2/11/44* at the same time point (two‐way ANOVA). dpi, days postinfestation. Bars, (a, b) 100 μm; (c, d) 50 μm.

The apoplastic sucrose that is taken up by the parasite has to be provided by the host plant through unloading from the host phloem. To identify the corresponding protein that releases sucrose from the host phloem, we screened an RNA‐seq dataset created from plant material during the interaction between the parasite *C. japonica* and its host *Glycine max* (Ikeue *et al*., 2015). We searched for upregulated Clade III SWEETs, known to function as bidirectional sucrose transporters and to be required for phloem unloading. By comparing upregulated *G. max* transcripts with the *A. thaliana* genome, we identified AtSWEET15 as a likely candidate for mediating sucrose efflux from the host phloem to the apoplast at the infection site. To prove that *AtSWEET15* exhibits enhanced expression levels during *C. reflexa* infection, we conducted qRT‐PCR on *Cuscuta*‐infected *A. thaliana* Col‐0 stem tissue. The analyses confirmed that the expression of *AtSWEET15* is significantly induced from 5 to 7 dpi, peaking at 6 dpi (Fig. 4g). To further analyze the function of AtSWEET15 during the infection, *A. thaliana* marker lines expressing *pAtSWEET15::gAtSWEET15‐GUS* (β‐glucuronidase) were infected with *C. reflexa*. We selected 8 dpi as a time point to observe the GUS staining to achieve the most intense signal, as GUS accumulates at the infection site. The GUS staining of the sections led to an intense GUS signal in the phloem adjacent to the invading haustorium (Fig. 4a). By contrast, no GUS activity was detectable in phloem bundles of the inflorescence distant to the penetrating haustorium (Fig. 4b,d). A more precise localization of GUS activity became visible in thin sections of the stained infection sites, which revealed GUS staining in the phloem of *A. thaliana* adjacent to the intruding haustorium (Fig. 4c). The results indicate that the penetrating haustorium of *C. reflexa* induces the expression of *AtSWEET15* in the host phloem.

*AtSWEET15* expression has been described to be regulated by S_1_‐bZIP transcription factors, which are involved in regulating carbon distribution from source to sink tissue (Kreisz *et al*., 2024). To determine whether S_1_‐bZIPs are involved in *Cuscuta*‐induced *AtSWEET15* promoter activation, *bzip2/11/44* triple mutants were infected with *C. reflexa* and samples of the infection site were collected for cDNA preparation. The qRT‐PCR analyses revealed that *bzip2/11/44* triple mutants exhibit reduced *AtSWEET15* expression at the infection site at any observed time point (Fig. 4g). This indicates that bZIP2/11/44 are necessary for the *Cuscuta*‐dependent activation of *AtSWEET15* expression.

### *Cuscuta campestris* seedling growth is reduced on *atsweet15* mutant host plants

To examine the physiological relevance of AtSWEET15 during the intrusive phase of the infection, we studied esculin transfer and *Cuscuta* growth on *atsweet15* knockout plants. As AtSWEET15 is presumably only important during the intrusive phase, an early time point of 4 dpi was chosen for the experiments.

Esculin was applied to leaves of *A. thaliana* Col‐0 and *atsweet15* knockout plants, and esculin fluorescence was measured in the attached *C. reflexa* haustoria at 4 dpi (Fig. 4e). Of the haustoria that were attached to Col‐0, 66.8% showed esculin fluorescence. By contrast, only 5.3% of the examined haustoria attached to *atsweet15* knockout plants showed esculin fluorescence, which is significantly less (*P* = 0.023) compared with the results from Col‐0 plants.

To determine the potential effect of AtSWEET15‐mediated sugar supply for *Cuscuta*, we infected *atsweet15* knockout plants with *C. campestris* seedlings and determined seedling fresh weight after 4 d of growth. The growth assay was performed with freshly germinated *C. campestris* seedlings because they have very limited energy reserves and the success of their initial attachment to a host is critical for their survival. The growth assay revealed that *C. campestris* seedlings grew significantly less (*P* = 0.0037) on *atsweet15* knockout plants compared with *A. thaliana* Col‐0 host plants. *C. campestris* seedlings infecting *atsweet15* knockouts reached a mean fresh weight of 2.4 mg, whereas seedlings grown on *A. thaliana* Col‐0 reached a mean fresh weight of 3.3 mg (Figs 4f, S4).

Both experiments indicate that AtSWEET15 has a high relevance during the early infection process as loss of AtSWEET15 in the host leads to impaired growth of *C. campestris* seedlings. This is likely caused by reduced sucrose uptake which is indicated by the reduced esculin uptake from the *atsweet15* mutants.

## Discussion

*Cuscuta* spp., like other holoparasitic plants, evolved strategies to withdraw phloem sap from host plants through symplastic connections (Haupt *et al*., 2001b; Birschwilks *et al*., 2006). However, establishing direct phloem connections to the host takes several days, and the development of haustoria is an energy‐consuming process. Germinated *Cuscuta* spp. seedlings desperately need to find a host before their limited energy reserves are depleted (Vaughn, 2002, 2003).

**Fig. 5:**
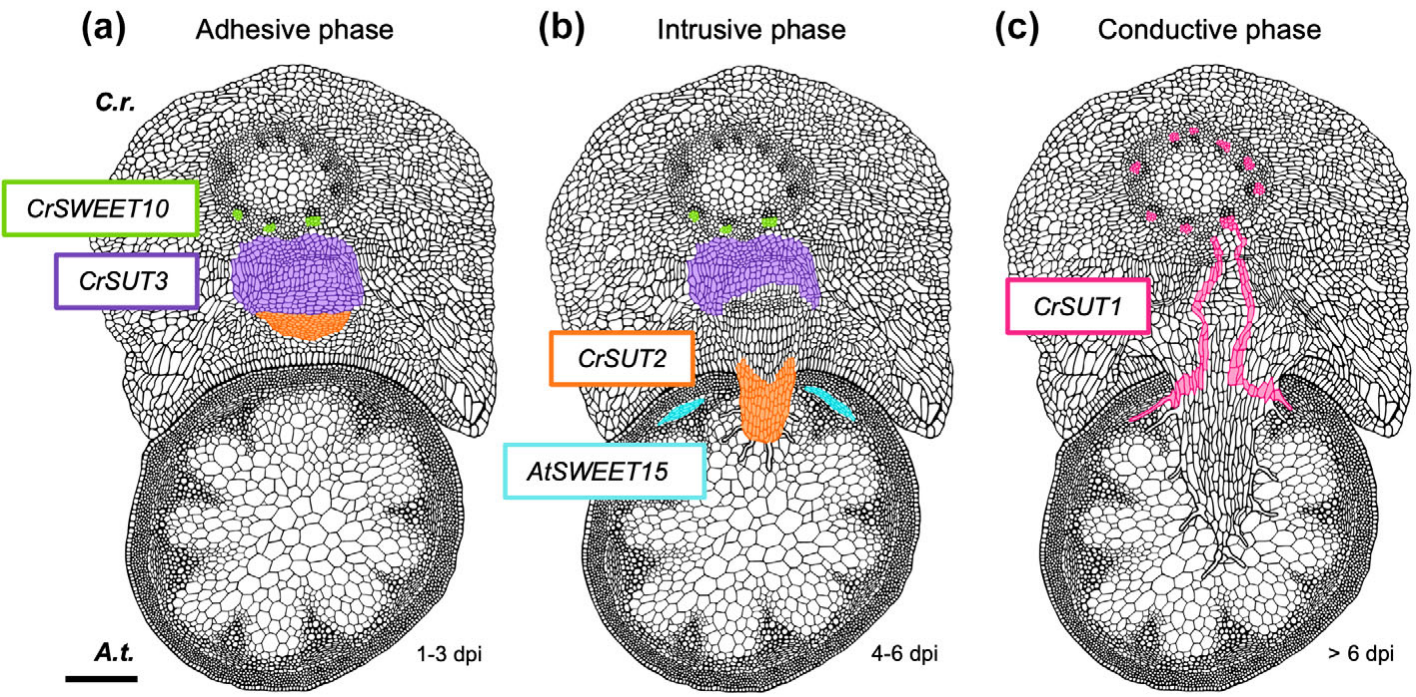
Schematic overview of sucrose transporter expression patterns during different infection stages. The tissue‐specific expression of SUGARS WILL EVENTUALLY BE EXPORTED TRANSPORTERS (*SWEET*) and SUCROSE TRANSPORTERS (*SUT*) genes is highlighted in color. (a) Adhesive phase: *CrSWEET10* is expressed in *Cuscuta reflexa* (*C.r.*) companion cells facing the infection site; *CrSUT3* is expressed in prehaustorial cells. (b) Intrusive phase: *CrSUT2* is expressed at the tip of the intruding haustorium; *AtSWEET15* is expressed in the *Arabidopsis thaliana* (*A.t.*) phloem adjacent to the intruding haustorium. (c) Conductive phase: *CrSUT1* is expressed in newly formed haustorial phloem. dpi, days postinfestation. Bar, 250 μm.

We analyzed *C. reflexa* and *A. thaliana* sucrose transporters across different phases of infection and identified four sucrose transporter genes in *C. reflexa*, *CrSWEET10* and *CrSUT1* to *3*, that were upregulated during the infection process. We demonstrated that all observed *C. reflexa* sucrose transporters recognize sucrose as a substrate, as shown by esculin uptake assays. In some cases, SWEET sucrose transporters are also capable of transporting glucose, such as AtSWEET9 (Lin *et al*., 2014), and this possibility cannot be excluded for CrSWEET10. However, given the companion cell‐specific localization of *CrSWEET10* mRNA, glucose transport was not relevant for this study, as the phloem sap of *A. thaliana* predominantly contains sucrose (Lohaus, 2022). One of the observed sucrose transporters, *CrSUT1*, was expressed in companion cells of the parasite shoot. In *A. thaliana*, knockout mutants were used to demonstrate that the companion cell‐specific sucrose H^+^ cotransporter AtSUC2 is responsible for phloem loading (Gottwald *et al*., 2000). The presence of *CrSUT1* mRNA in companion cells of *C. reflexa* strongly suggests that CrSUT1 also functions as the protein responsible for the loading of sucrose into the phloem. Even though holoparasitic plants do not produce their own assimilates, they rely on mechanisms for phloem loading and sucrose retrieval. This was previously observed in the root holoparasite *Phelipanche ramosa*, where expression of phloem loading *PrSUT1* was detected in the phloem (Péron *et al*., 2016). *CrSUT1* expression during the infection is related to the phloem development of the intruding haustorium, when phloem cells differentiate from haustorial parenchyma cells. The resulting daughter cells mature into a sieve element and a companion cell (Dörr, 1990). We further examined the spatiotemporal expression and function of *CrSWEET10*, *CrSUT2* and *CrSUT3* (summary Fig. 5).

Upon initial host contact, the cortical cells of the *C. reflexa* stem oriented toward the host, differentiate into prehaustorial cells, dividing rapidly with high metabolic activity (Lee, 2007). This newly formed meristem represents a strong sink for the parasite and the expression levels of *CrSWEET10* and *CrSUT3* are tissue‐specifically elevated. These findings suggest that the developing haustorial meristem is supplied with sucrose by CrSWEET10‐mediated unloading from companion cells. Sucrose is subsequently taken up from the apoplast by CrSUT3 into prehaustorial cells. In photoautotroph plants, such as *A. thaliana* or *N. tabacum*, symplastic assimilate transfer is the preferred strategy for supplying sink tissues with soluble sugars. Tracer studies in *A. thaliana* revealed that unloading occurs symplastically in sink leaves, shoot and root apical meristems, flowers, anthers and ovule primordia (Oparka *et al*., 1994; Gisel *et al*., 1999; Imlau *et al*., 1999; Stadler *et al*., 2005; Werner *et al*., 2011). A similar pattern has been observed in *N. tabacum* and barley, where CF is unloaded symplastically in sink leaves (Oparka *et al*., 1999; Haupt *et al*., 2001a). By contrast, the apoplastic unloading mechanism in the haustorial meristematic tissue of *C. reflexa* may be linked with its unique adaption to a heterotrophic lifestyle.

Once the prehaustorium is established, the intrusive phase is initiated. Prehaustorial cells elongate, penetrate the host tissue and searching hyphae grow out from the intruding haustorium to find the host vasculature (Yoshida *et al*., 2016). We demonstrate that esculin uptake into the haustorium occurs before a symplastic connection to the phloem. Additionally, our spatiotemporal expression analysis revealed that *CrSUT2* mRNA is abundant in the penetrating haustorium during the intrusive phase. Therefore, *C. reflexa* takes up sucrose from the host apoplast as soon as the haustorium starts to penetrate. This strategy enables the parasite to access host sucrose before the haustorium enters the conductive phase. In an evolutionary context, the apoplastic method of nutrient acquisition might represent an ancestral strategy and likely preceded the establishment of a symplastic nutrition, which may have evolved later as the haustorium became more specialized and efficient. It cannot be excluded that *Cuscuta* spp. acquire nutrients via symplastic connections with host parenchyma cells during the intrusive phase. However, it remains unclear whether plasmodesmata are present between *Cuscuta* searching hyphae and host parenchyma cells in the intrusive phase and whether they are functional. Moreover, such connections would likely contribute only marginally to nutrient uptake compared with the substantial transfer enabled by direct access to the host phloem.

The existence of sugar transporters that drive the interspecific apoplastic exchange of sugars between *Cuscuta* spp. and hosts has been postulated previously. Searching hyphae wrapped around host sieve tubes form membrane protruberances, which are typical for transfer cells with a strong demand for apoplastic nutrient transfer. This indicates the existence of sucrose transporters and the need for apoplastic sugar uptake into searching hyphae (Dörr, 1969, 1972). Based on our observations, sucrose is received from a broader area of the host apoplast. This is evident as esculin uptake from the host into the haustorium occurs even when a distinct distance remains between the searching hyphae and the host phloem.

SUT2/SUC3 sucrose transporters have a high sequence similarity across different species, characterized by their distinctive long cytoplasmic loop in the central region of the protein (Barker *et al*., 2000; Meyer *et al*., 2000; Barth *et al*., 2003; Eckardt, 2003). The expression of *SUT2/SUC3* is sink‐specific, as promoter activity has been detected in the pollen grains and tubes, guard cells, trichomes, root tips, lateral root tips, stipules and ovules (Meyer *et al*., 2004). Similarly, *CrSUT2* was found to be expressed in dividing sink tissues, including the haustorium and the apical meristem. Active apoplastic sucrose uptake via CrSUT2 into the *Cuscuta* haustorium necessitates a corresponding transporter responsible for sucrose unloading on the host side. In *A. thaliana*, *AtSWEET15* is induced upon infection with *C. reflexa* at the infection site adjacent to the intruding haustorium. AtSWEET15 belongs to Clade III SWEETs, which transport sucrose passively along a concentration gradient, and localizes to the plasma membrane (Seo *et al*., 2011). A synonym for *AtSWEET15* is *AtSAG29* (SENESCENCE‐ASSOCIATED GENE 29), as it is expressed in senescent organs (Quirino *et al*., 1999) and is also involved in seed filling (Chen *et al*., 2015). The relevance of AtSWEET15 for the infection process was shown as significantly less esculin was transferred from Arabidopsis *atsweet15* to *C. reflexa* haustoria compared with Col‐0 hosts. Additionally, a growth assay demonstrated that *C. campestris* seedlings gained significantly less weight and grew less on *atsweet15* mutants compared with *A. thaliana* Col‐0. By inducing the tissue‐specific expression of *AtSWEET15*, *C. reflexa* manipulates the host, probably by mimicking a sink. This process requires specific signaling pathways in the host and the control of gene expression. S_1_ basic leucine zipper (bZIP) transcription factors regulate carbon distribution from source leaves into sink tissues and several S_1_‐bZIPs control the tissue‐specific expression of some Clade III *AtSWEETs*, including *AtSWEET15*. For *AtSWEET15*, it was reported that specific G‐Box related elements in the promoter sequence are responsible for binding of bZIP11 and subsequent chromatin‐opening in the promoter region (Kreisz *et al*., 2024). Our results show that *bzip2/11/44* plants showed reduced *AtSWEET15* expression upon infection. Since the *Cuscuta*‐induced *AtSWEET15* expression is reduced rather than completely abolished in *bzip2/11/44*, identifying the additional TFs and regulatory mechanisms underlying this expression are of interest in the future. Investigations of specific signals and the downstream cascades that trigger *AtSWEET15* transcription via phloem‐specific bZIP2/11/44 activation will give insights to the mechanisms of new sink establishment.

It is commonly observed that plant pathogens hijack SWEETs to gain access to sugars from their hosts (Breia *et al*., 2021). In many microbial infections, *SWEET* gene expressions are upregulated (Chen *et al*., 2010) and are targets for resistance against pathogens. For instance, the secretion of a bacterial effector that binds to the *OsSWEET11* promoter and induces its transcription is crucial for a successful infection of rice by *Xanthomonas oryzae* pv *oryzae* (Chu *et al*., 2006; Yang *et al*., 2006; Chen *et al*., 2010). In *A. thaliana*, it has been shown that *Pseudomonas syringae* pv *tomato* DC3000 highly induces the expression of several *SWEET* genes, particularly *AtSWEET15*. Additionally, *AtSWEET15* is upregulated during *Botrytis cinerea* infection. Those pathogen‐specific modifications of *SWEET* mRNA levels likely result in increased sugar efflux from host cells at the infection sites, which in turn promotes pathogen growth (Chen *et al*., 2010). Furthermore, it was shown that increased levels of various host sugar transport proteins, including SWEETs, are important for the infection process of root–knot nematodes (Zhou *et al*., 2023; Sun *et al*., 2024). Giant cells of root–knot nematodes remain symplastically isolated and rely on apoplastic loading mechanisms (Hoth *et al*., 2008). Therefore, hijacking *SWEETs* of host plants seems to be a highly important and common strategy for successful infections by various pathogens and herbivores. It would be interesting to investigate whether host plants, as a defense strategy against *Cuscuta*, express genes encoding monosaccharide transporters and extracellular invertases to recover the apoplastic sucrose exported by AtSWEET15. It has been shown for various microbial plant pathogens that the induction of monosaccharide transporters and invertase genes confers resistance (Fotopoulos *et al*., 2003; Lemonnier *et al*., 2014; Proels & Hückelhoven, 2014; Yamada *et al*., 2016; Chen *et al*., 2023). In the future, it may also be interesting to investigate whether *Cuscuta* spp. induce the expression of invertases in the host and can import hexoses via monosaccharide transporters.

The ability of *Cuscuta* spp. to hijack a SWEET‐mediated efflux pathway in the stems of their host plants makes them a great model for studying the mechanisms behind sugar allocation in crop plants. As *Cuscuta* spp. have a very wide host spectrum, they likely take advantage of a general mechanism to alter sucrose allocation according to their needs, which must function across various plant species. Gaining insights to this process could help improve the carbon distribution into fruits or storage organs, ultimately leading to higher yields in crops.

## Competing interests

None declared.

## Author contributions

MH, RS and MA designed the experiments. MH, PS, RB, AG and DE carried out the experiments. MH, FK and RS designed the experiments related to sugar transport measurements with radionuclides. MH, RS, US and MA discussed the results. MH, RS, IA and MA wrote the manuscript. RS and MA share senior authorship of this work. All coauthors contributed to the revision of the manuscript and approved the final manuscript.

## Disclaimer

The New Phytologist Foundation remains neutral with regard to jurisdictional claims in maps and in any institutional affiliations.

## Supporting information


**Fig. S1** Uptake of fluorescent tracers into haustoria of *Cuscuta reflexa*.
**Fig. S2** Phylogenetic analyses of sucrose transporter amino acid sequences.
**Fig. S3** *In situ* hybridization of *CrSUT2* and *CrSUT3* mRNA.
**Fig. S4** Additional data on the growth assay of *Cuscuta campestris* seedlings on *Arabidopsis thaliana* Col‐0 and *atsweet15*.
**Table S1** Known sequences of *CrSWEET10*, *CrSUT1*, *CrSUT2* and *CrSUT3* cDNA.
**Table S2** Primer sequences used for qRT‐PCRs, cloning and probe synthesis for *in situ* hybridization.
Please note: Wiley is not responsible for the content or functionality of any Supporting Information supplied by the authors. Any queries (other than missing material) should be directed to the *New Phytologist* Central Office.

## Data Availability

The data that support the findings of this study are available in the article and Figs S1–S4, Tables S1 and S2
