# Supplementary material for: Dodder seedlings benefit from apoplastic nutrition by inducing SWEET15‐mediated unloading of sucrose from the host's phloem
Source: New Phytol. 2026 May 16;252(2):829–42. doi: 10.1111/nph.71270 (PMC13583392; doi:10.1111/nph.71270)
Supplement: Supplementary file 1 [file NPH-252-829-s001.pdf]

New Phytologist

***New Phytologist* Supporting Information**

Article title: Dodder seedlings benefit from apoplastic nutrition by inducing SWEET15-mediated unloading of sucrose from the host's phloem

Authors: Maleen Hartenstein, Dominik Eydam, Antonia Klatt, Arne Greif, Peter Slaby, Ronja Burggraf, Franz Klebl, Isabell Albert, Uwe Sonnewald, Ruth Stadler, Markus Albert

Article acceptance date: 29 April 2026

The following Supporting Information is available for this article:

Fig. S1 Uptake of fluorescent tracers into haustoria of C. reflexa. (A, B) Sections of an artificially induced haustoria of *C. reflexa* without host contact. Prior to sectioning, stems with haustoria were incubated with solutions containing membrane permeable CFDA (A) and membrane impermeable fluorescein (B). (C) *pAtSUC2::GFP* in *N. tabacum* (*N. t.*) infected with *C. reflexa* 6 dpi. *N. tabacum* source leaves were loaded with esculin. Arrowheads indicate GFP fluorescence in the host phloem. (D) *pAtSUC2::GFP* in *N. tabacum* infected with *C. reflexa*, 14 dpi. GFP fluorescence is visible in the haustorium and *C. reflexa* vasculature. (E) *N. tabacum* infected with *C. reflexa*, 8 dpi. *N. tabacum* source leaves were loaded with CFDA. CF fluorescence is visible in the haustorium. Scale bars: 250 µm in (A, B), 300 µm in (C-E).

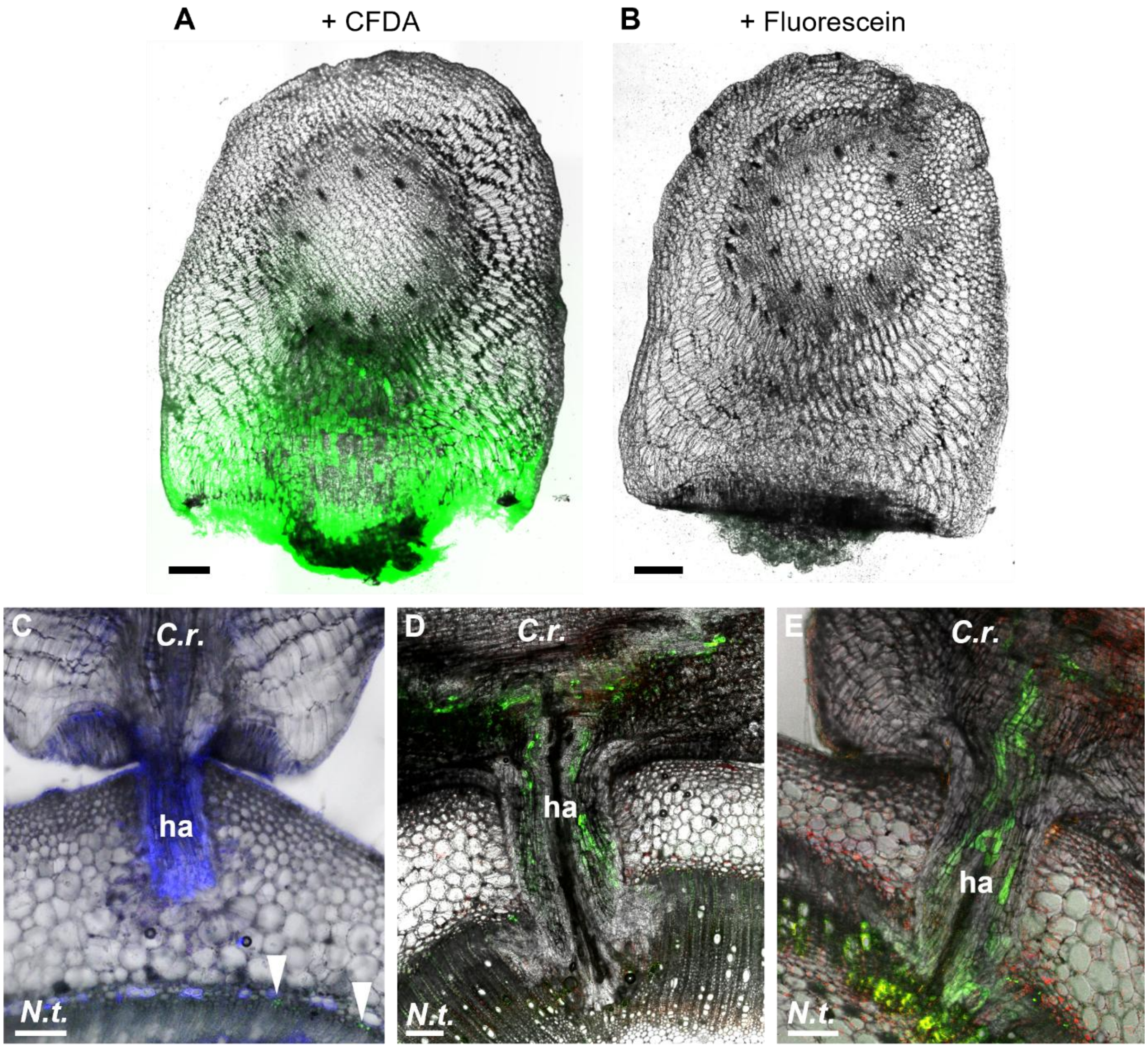

A
+ CFDA
B
+ Fluorescein
C
C.r.
ha
N.t.
D
C.r.
ha
N.t.
E
C.r.
ha
N.t.

Fig. S2 Phylogenetic analyses of sucrose transporter amino acid sequences. (A) Phylogenetic tree of various SUT protein sequences, including CrSUT1, CrSUT2 and CrSUT3 (red boxes) (B) Phylogenetic tree of various SWEET protein sequences, including CrSWEET10 (red box). The evolutionary analyses were conducted with MEGA11 (Tamura et al., 2021). The sequences were aligned with ClustalW. The Maximum Likelihood method and Le_Gascuel_2008 model were used (Le & Gascuel, 2008). Bars indicate evolutionary distance (substitutions per site). Accession numbers of SUT protein sequences in (A): AtSUC1 (*Arabidopsis thaliana*; AT1G71880), AtSUC2 (*A. thaliana*; AT1G22710), AtSUC3 (*A. thaliana*; AT2G02860), AtSUC4 (*A. thaliana*; AT1G09960), AtSUC5 (*A. thaliana*; AT1G71890), AtSUC6 (*A. thaliana*; AT5G43610), AtSUC7 (*A. thaliana*; AT1G66570), AtSUC8 (*A. thaliana*; AT2G14670), AtSUC9 (*A. thaliana*; AT5G06170), CsSUT1 (*Citrus sinensis*; KAH9757961), CsSUT4 (*C. sinensis*; KAH9755980), FsSUT1 (*Fagus sylvatica*; AYU70941), IbSUT1x (*Ipomoea batatas*; ADL63119), IbSUT2x (*I. batatas*; ADL63120), IbSUT2y (*I. batatas*; ADL63121), IbSUT3 (*I. batatas*; QGW08804), IbSUT4 (*I. batatas*; QGW08803), NtSUT1 (*Nicotiana tabacum*; AUD40045), NtSUT2 (*N. tabacum*; BBN23602), NtSUT3 (*N. tabacum*; AAD34610), NtSUT4 (*N. tabacum*; BBN23601), OsSUT1 (*Oryza sativa*; AAF90181), OsSUT2 (*O. sativa*; AAN15219), OsSUT3 (*O. sativa*; BAB68368), OsSUT4 (*O. sativa*; BAC67164), OsSUT5 (*O. sativa*; BAC67165), PaSUT1 (*Picea abies*; AWW87343), PaSUT2 (*P. abies*; AWW87344), PfSUT2 (*Perilla frutescens* var. *hirtella*; KAH6756336), PfSUT4 (*P. frutescens* var. *hirtella*; KAH6776400), PpSUC3 (*Physcomitrium patens*; XP_024382624), QrSUT1 (*Quercus robur*; AHB33871), QrSUT2 (*Q. robur*; AWW87345), QrSUT4 (*Q. robur*; AYU70940), SlSUT1 (*Solanum lycopersicum*; NP_001289830), SlSUT2 (*S. lycopersicum*; AAG12987), SlSUT4 (*S. lycopersicum*; AAG09270), StSUT1 (*Solanum tuberosum*; CAA48915), StSUT2 (*S. tuberosum*;

AAP43631), StSUT4 (*S. tuberosum*; AAG25923),VvSUC11 (*Vitis vinifera*; AAF08329), VvSUC12 (*V. vinifera*; AAF08330), VvSUC2 (*V. vinifera*; RVW94479), VvSUC27 (*V. vinifera*; ADP37123), VvSUC3 (*V. vinifera*; RVW82393). Accession numbers of SWEET protein sequences in (B): AtSWEET1 (*A. thaliana*; AT1G21460), AtSWEET2 (*A. thaliana*; AT3G14770), AtSWEET3 (*A. thaliana*; AT5G53190), AtSWEET4 (*A. thaliana*; AT3G28007), AtSWEET5 (*A. thaliana*; AT5G62850), AtSWEET6 (*A. thaliana*; AT1G66770), AtSWEET7 (*A. thaliana*; AT4G10850), AtSWEET8 (*A. thaliana*; AT5G40260), AtSWEET9 (*A. thaliana*; AT2G39060), AtSWEET10 (*A. thaliana*; AT5G50790), AtSWEET11 (*A. thaliana*; AT3G48740), AtSWEET12 (*A. thaliana*; AT5G23660), AtSWEET13 (*A. thaliana*; AT5G50800), AtSWEET14 (*A. thaliana*; AT4G25010), AtSWEET15 (*A. thaliana*; AT5G13170), AtSWEET16 (*A. thaliana*; AT3G16690), AtSWEET17 (*A. thaliana*; AT4G15920), OsSWEET1a (*O. sativa*; Q8RZQ8), OsSWEET1b (*O. sativa*; Q60EC2), OsSWEET2b (*O. sativa*; Q5N8J1), OsSWEET3a (*O. sativa*; Q0DJY3), OsSWEET4 (*O. sativa*; Q6K4V2), OsSWEET5 (*O. sativa*; Q6L568), OsSWEET6a (*O. sativa*; Q8LR09), OsSWEET6b (*O. sativa*; Q8W0K2), OsSWEET7a (*O. sativa*; Q0J361), OsSWEET7b (*O. sativa*; Q0J349), OsSWEET7e (*O. sativa*; A3BWJ9), OsSWEET11 (*O. sativa*; NP_001390599), OsSWEET12 (*O. sativa*; A2XGM7), OsSWEET13 (*O. sativa*; Q2QR07), OsSWEET14 (*O. sativa*; Q2R3P9), OsSWEET15 (*O. sativa*; Q6K602), OsSWEET16 (*O. sativa*; Q10LN5), VvSWEET1 (*V. vinifera*; RVW85053), VvSWEET2a (*V. vinifera*; RVW79000), VvSWEET3 (*V. vinifera*; RVW26723), VvSWEET4 (*V. vinifera*; RVW14880), VvSWEET5 (*V. vinifera*; RVW52868), VvSWEET10 (*V. vinifera*; RVW52674), VvSWEET12 (*V. vinifera*; RVW52673), VvSWEET14 (*V. vinifera*; RVW54985), VvSWEET15 (*V. vinifera*; RVW84085), VvSWEET16 (*V. vinifera*; XP_002279031), VvSWEET17 (*V. vinifera*; RVW60370).

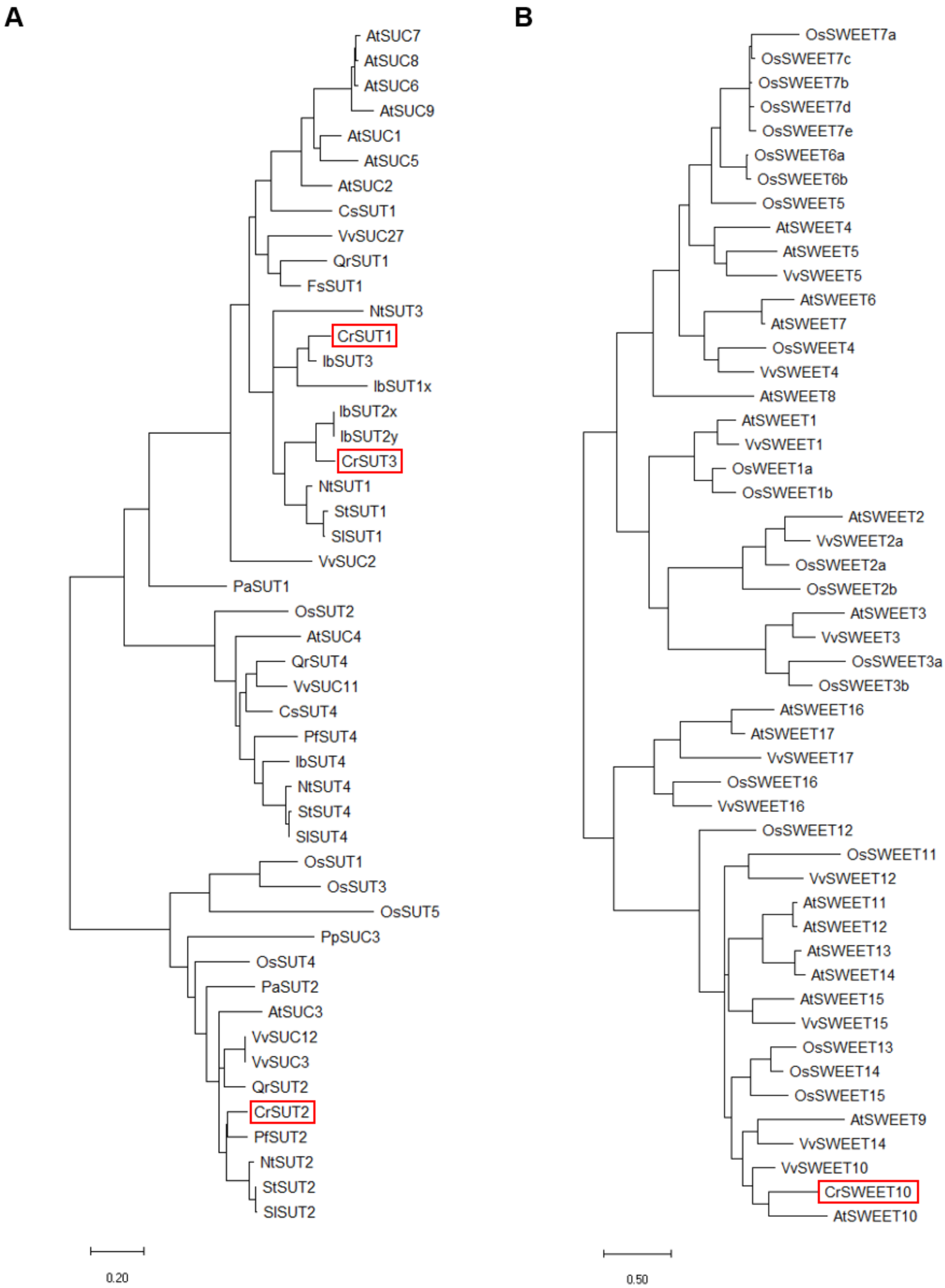
A
AtSUC7
AtSUC8
AtSUC6
AtSUC9
AtSUC1
AtSUC5
AtSUC2
CsSUT1
VvSUC27
QrSUT1
FsSUT1
NtSUT3
CrSUT1
IbSUT3
IbSUT1x
IbSUT2x
IbSUT2y
CrSUT3
NtSUT1
StSUT1
SlSUT1
VvSUC2
PaSUT1
OsSUT2
AtSUC4
QrSUT4
VvSUC11
CsSUT4
PfSUT4
IbSUT4
NtSUT4
StSUT4
SlSUT4
OsSUT1
OsSUT3
OsSUT5
PpSUC3
OsSUT4
PaSUT2
AtSUC3
VvSUC12
VvSUC3
QrSUT2
CrSUT2
PfSUT2
NtSUT2
StSUT2
SlSUT2
0.20
B
OsSWEET7a
OsSWEET7c
OsSWEET7b
OsSWEET7d
OsSWEET7e
OsSWEET6a
OsSWEET6b
OsSWEET5
AtSWEET4
AtSWEET5
VvSWEET5
AtSWEET6
AtSWEET7
OsSWEET4
VvSWEET4
AtSWEET8
AtSWEET1
VvSWEET1
OsWEET1a
OsSWEET1b
AtSWEET2
VvSWEET2a
OsSWEET2a
OsSWEET2b
AtSWEET3
VvSWEET3
OsSWEET3a
OsSWEET3b
AtSWEET16
AtSWEET17
VvSWEET17
OsSWEET16
VvSWEET16
OsSWEET12
OsSWEET11
VvSWEET12
AtSWEET11
AtSWEET12
AtSWEET13
AtSWEET14
AtSWEET15
VvSWEET15
OsSWEET13
OsSWEET14
OsSWEET15
AtSWEET9
VvSWEET14
VvSWEET10
CrSWEET10
AtSWEET10
0.50

Fig. S3 In situ hybridization of *CrSUT2* and *CrSUT3* mRNA. (A, B) Sections of *C. reflexa* infecting *Coleus blumei* in the intrusive phase, hybridized with *CrSUT3* antisense (A) and sense (B) probes. (C, D) Sections of C. reflexa apical meristem, hybridized with *CrSUT2* antisense (C) and sense (D) probes. Scale bars: (A, B) 500 µm; (C, D) 200 µm.

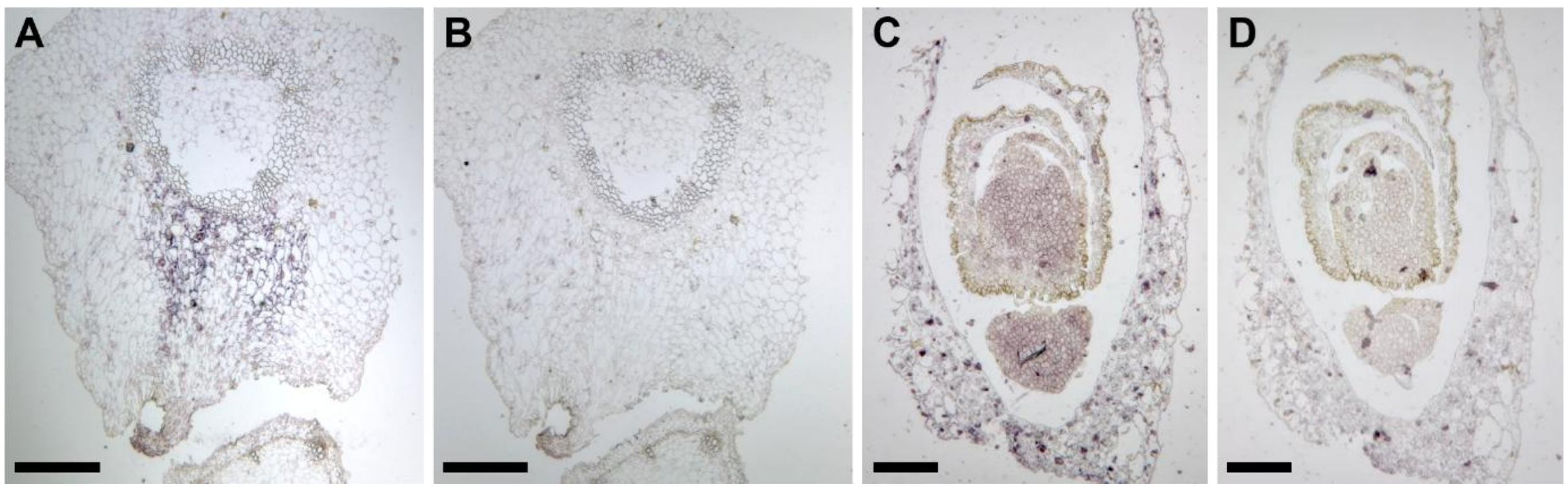

Fig. S4 Additional data on the growth assay of *C. campestris* seedlings on *A. thaliana* Col-0 and *atsweet15*. (A) and (B) show two independent repetitions of the growth assay in Fig. 4F. The box plots show the means of the fresh weight of the seedlings at 4 dpi. Red dots show independent values. (A) *A. thaliana* Col-0 (n = 8) and *atsweet15* (n = 8). (B) *A. thaliana* Col-0 (n = 4) and *atsweet15* (n = 8). The p-values were calculated using a one-tailed Welch's t-test and are displayed above the graphs.

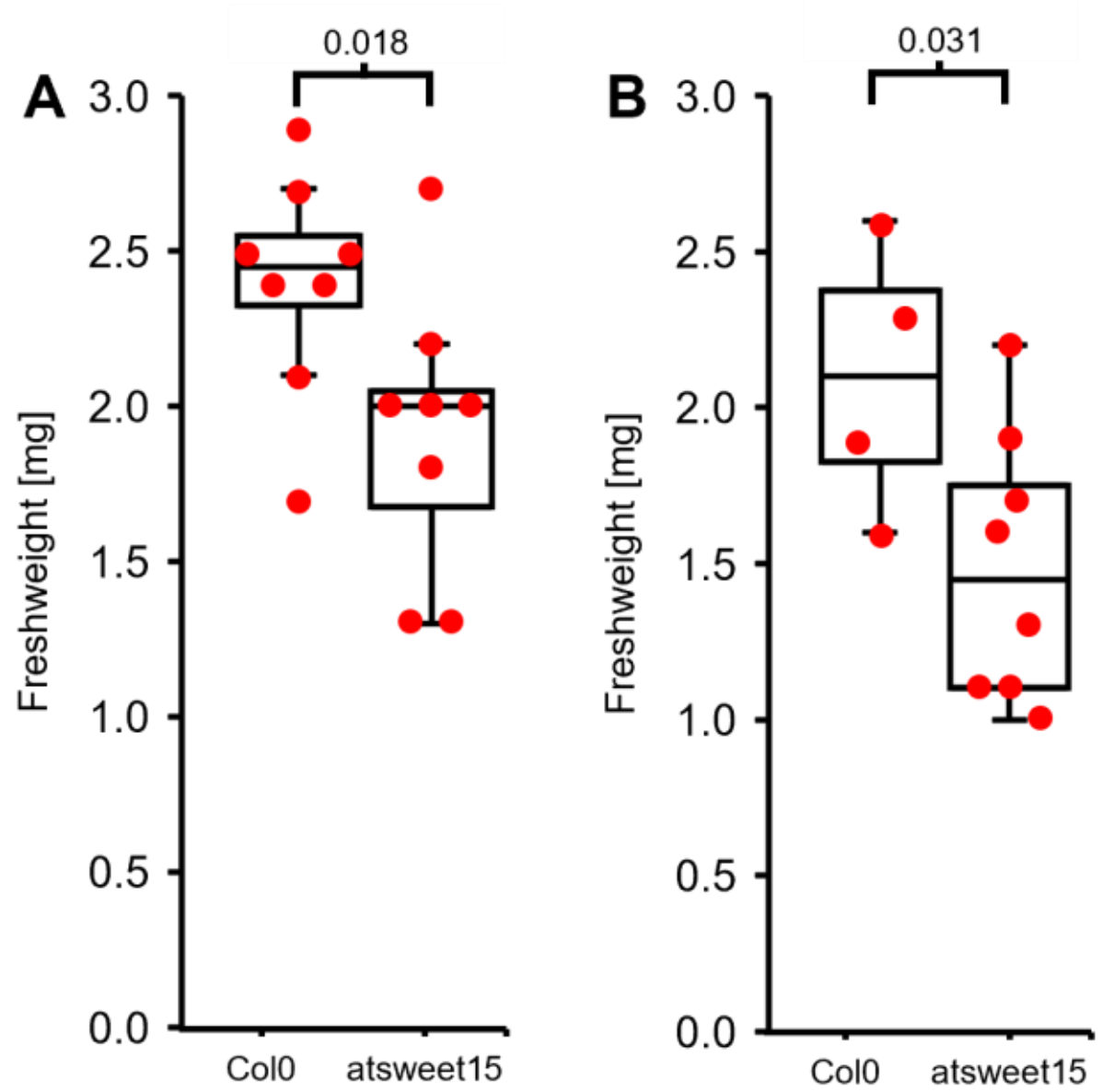

Table S1 Known sequences of *CrSWEET10*, *CrSUT1*, *CrSUT2* and *CrSUT3* cDNA. The coding sequence is shown in gray.

**Gene**	**Sequence**
CrSWEET10	AGTTATTTCATCTCTCTCTACAAAACATATACATTTAGAGAGAGAGAGAGAGATTT<br>CTTAGGTTCTACAAAGAAAAAAGCACCATGGCTTTCTCTGGTCATAACTGGGCTTT<br>TGTTTTTGGTGTTCTTGGAAACATCATCTCATTCATTGTCTTCCTTTCTCCACTACCA<br>ACATTCTATCAGATGTACAAGAAGAAATCAACTGAAGGATATCAATCAATTCCTTA<br>TGTGGTTGGACTATTCAGTGCCATGCTTTGGATATACTATGCATTTGTCAAAACCT<br>CAAACACCACTCTTCTCATCACTATCAACACCTTTGGTTGTTTCATTGAGACCATTT<br>TCATTGTCTTCTTCCTCTACTATTCAACCAAGAAAACAAGGATACAAACAATCAAG<br>TTTCTTGCTTTGTTTGTGGCTGGAGGGTTTGGTGCTATTGTTCTTGTCACACACTTT<br>CTATTCAAAGCCCCATTTCGTGCCGAAGTTGTTGGATGGATTTGCCTCGTGTTTTC<br>CTTGTGTGTGTTCGTCGCCCCCCTTTGCATAGTGAGACAAGTGATTAAAACCAAG<br>AGTGTTGAAAACATGCCAGTGCTCCTCTCAGTTTTCCTCACCTTCAGTGCTGTGAT<br>GTGGTTCTTCTATGGCTTGTTTCAAAAAGACATGAACATTGCTGTGCCAAATGTTT<br>TGGGCTTCTTCTTTGGAATTCTTCAAGTTGGTCTCTACATGGTGTACAAGGACAAG<br>AAGAAAGAGATAGTGAAGGAGCAAAGGCTTCCTGAAGTGGCACTATCAAAACCA<br>GTTATAATCTTGGAAGATGAGACTAACACTAATGAGAACAACAACAGGCACAAG<br>CTTCCAGAGCTCACTGAGGAACAAATCATCGACATCGTCAAGCTCGGATCACTCG<br>TGTGTTCGGACAAAATTCCAAAGAATGGAGTTGATATCGAGGCTGCAGCTCCTAA<br>GCTTCAAGCTGTGGAAGCTTGAGAAGAAGATAATAATCAAGATATAGTATATATA<br>TACACATATTAATATATAAATGTATGTGGTGAGAGTATTAATTTGGCTGGATTTCC<br>TCTGGCTGGTGTTATCAGTTATGTATGTAACATGTCTACCCTTACTTTGTACACGTA<br>TGGGTGCTAATGGATTTGGT
CrSUT1	TTTTCCCACTTCCCTTTTACAATCACAATTATATCCTCTCACTAGACTCTATGGAGA<br>CAGCCTCTGCTAACGACAAATCGTCCTTTCAAGTGGAGGAAGCTTCCCCGGCGGC<br>ACAAACGCCGATATGGAAGATAGTGCTGGTGGCTTCCATCGCCGCCGGTGTCCA<br>GTTCGGGTGGGCGTTACAGCTGTCCCTCCTTACCCCTTATGTCCAGCTGCTTGGAG

	TCCCGCACAAGTTCGCCTCCTTCATGTGGCTTTGCGGCCCCATCTCCGGCATGATC<br>GTCCAGCCGCTTGTCGGCTACTACAGCGACAACTGTACTTGGCGCTTCGGCCGCC<br>GTCGCCCCTTCATTGCTTCCGGTACTTTGCTTGTCATTCTTGCCGTCTTTCTCATTG<br>GGTTCGCGGCTGATATTGGTTACGCCGCCGGCGACCTCCTCGAGAAAACCACCAA<br>GCCCCGGTCTGTCGTTGTTTTTGCGGTTGGGTTTTGGGTCCTTGATGTTGCCAATA<br>ACATGTTGCAGGGCCCGTGCAGGGCACTATTGGCTGATCTCTCCGGAGGGAGTG<br>CCGACAGAATGCGGGCTTCAAACGCGCTGTTCTCGTTTTTCATGGCGATAGGAAA<br>CGTCCTTGGCTACGCCGCCGGCTCCTACTCCGGCCTCCATAAAGTCTTCCCCTTTTC<br>AAAGACTAAAGCTTGCGACCTCTACTGCGCCAATCTCAAGAGCTGTTTCTTCATCT<br>CTGTGGCTCTTTTGCTCACAGTGACCACCTTGGCGCTCACGTTTGTCAAAGAGCAA<br>GTGGTCAAAGACGTCGCCGACGACGGAGAAAAGGGGACGGCGAAGGTGCCGGT<br>CTTCGGGGAAATCTTCGGCGCCCTGAAGGACCTCCCTAGGCCGATGTGGATACTC<br>TTACTCGTCACTGCCCTGAACTGGATGGCGTGGTTTCCTTTCTTGCTCTACGACAC<br>CGATTGGATGGCCAAGGAGGTGTACGGCGGAAAAGTCGGCGACAGATTATACG<br>CCAGAGGCGTCCATTCCGGCGCGCTCGGTCTTATGCTTAATTCTGTCGTTTTAGGC<br>TTCACGTCAATAGCGGTGGAGCTCCTGGCACGGCGGCTCGGCGGAGTGAAGAAG<br>CTCTGGGGCGGTTGCAACTTCATCTTGGCTATTTCCTTGGCCATGACAGTTTTGGT<br>CACCAAAATGGCGCAGCATTCACGGCCGCACGACGCCGGCGGCAACATTCTTCCC<br>CCCACCGCCGGTGTTAAGGCCGGAGCCTTGACTCTATTCTCCGTCCTCGGGATTCC<br>TCTGGCCGTTACTTTTAGCATCCCATTCGCTCTGGCATCTATCTTTTCGAGCAACGC<br>AGGAGCTGGCCAAGGTCTATCATTAGGCGTCCTCAACCTTTCAATTGTCATCCCGC<br>AGATGCTGGTGTCGTTTTTGAGTGGGCCGTGGGACGCGCTGTTTGGCGGTGGGA<br>ATTTGCCGGCGTTTGTGGTGGGAGCCGTCTGTGCGGCGGCGAGTGGGATTTTTG<br>CTATGACTTTGCTTCCGTCTCCGCCGCGTGCGGTGAATCTCGGGTTATCTGCGGCC<br>GGAGGTTTCCATTGAAACCGGTGATTTTGGTTTTGGGTTTCCTTGTTAAGAGAAAT<br>ATAGAAAACCTGCCTCCCATGTGGTCTGCTATTTGTCTGACCAATTTGGAGCTATT<br>AAGAATATGTTTGGATGGAATGTAAATGAGTGTAATAATGGAAGTGTAAGTAAT<br>ACTACTCTATAAAGTGAGTTGAATTAAAGTCATACTTGGTGTATGTGTATGTTTAC

	CTTATTCTTGTTTGGGATGACAAATGAGAAAAGAAAAATGTAAATAATTACTATG<br>ATTATTTTAATCTAAGTAAATCAATGAGNGNTGAAAAGT
CrSUT2	CAAACTCAGCGCGCCCAAATTTTGATCTGTTGATCTTCTGCTTCTTCTCTATCATTA<br>CACCTATTCTCTCACTAAAACCTCCATTTGGATGAATCACTTCGGTTAGACTAAATT<br>CATTTCATAATAAGTAATAATTTGTATACATCTGAATTGCTCCTTCGGAAAAGTTTC<br>ACAGTTTTGCACGCATCTGGATGTAAAAAAATTGTACCTAGATTGACTGGATCAG<br>GTGAAGGAGCGGTGGGCAGGGGGTGGAGATGATGGATGCGGTGTCGATCAGG<br>GTGCCATATAAGAATTTAAAGCAGGAGGTGGAGCTGGTAGGGGTAGACGAGCTT<br>CATCTGCGTCGGTTGCAGGTAGATTCGCCGCCGTCACCTCCTAGGGATTCCAATG<br>GCGACTCCACGGATTCGAATCCTCACCAGCCGCCTGCGCACGTCAACCTACTCACC<br>TTGATCCTCAGTTGCACGGTCGCAGCAGGTGTCCAATTTGGCTGGGCGTTGCAAC<br>TCTCTCTCCTCACCCCTTATATTCAGACTCTTGGCATACAACATGCCTTCTCTTCCTT<br>TATTTGGCTATGTGGCCCAATTACAGGCCTTGTGGTGCAACCTTGTGTTGGAATAT<br>GGAGTGACAAATGCTCTTCCAAATACGGCAGAAGAAGGCCTTTTATATTTGTTGG<br>ATCTCTCATGATCTCCTTTGCTGTAATAATAATTGGATTTTCTGCAGACATAGGATA<br>CATTATAGGTGACACGAAAGAGCACTGCAGCACTTTCAAAGGTACTCGTACAAGG<br>GCAGCTGTTGTGTTCATCATTGGATTTTGGCTGTTGGATCTCGCAAACAATACTGT<br>ACAGGGCCCAGCTCGAGCTCTTTTGGCTGACTTATCAGGTCCTGAGCAACGAAAT<br>ACTGCAAACGCTATATTTTGCTCCTGGATGGCTGTTGGGAATATTCTAGGATTTTC<br>TGCTGGAGCTAGTGGAAATTGGCACAGATGGTTTCCTTTCCTGATGAGCAGAGCA<br>TGCTGCGAGGCATGTGGGAACCTCAAGGCAGCATTTTTAGTCGCTGTGGTGTTTC<br>TGGCTCTGTGTACTCTAATAACTCTCTACTTCGCCAAGGAAGTCCCACTGACGGCT<br>GTGCAATTTCACCGGTTGTCAGATTCTTCTCCTCTCTTGGATAGTCCTCCGGATACT<br>GGATTAGATCTTTCACACCCAAAAAACGACACACAGCCCCTCAATATTATTTCTGT<br>CAACAAATCCGAGAATGGTTATATGGATAATAATCTGAAAAATGAAGACCAAAAC<br>GGAGGGAATGATCCAGCTGATAGCTTTAATGATGGTATTGGTGCAGTTTTGGTTA<br>ATCTATTGACCAGCTTAAGACATTTGCCTCCTGCAATGCATTCAGTGCTCGCTGTC<br>ATGGCATTGACGTGGTTGTCTTGGTTTCCCTTTTTCCTTTTTGACACTGATTGGATG<br>GGAAGAGAAGTCTATCATGGGGATCCAAATGGAGATGTTTCTGAAGTAAAAACT

	TACAATCAAGGTGTCAGAGAAGGTGCATTTGGTTTGCTATTGAATTCTGTTGTGCT<br>TGGCATAACATCTTTCTTTATCGATCCAATGTGCCAGTGGATTGGTGCAAGACTGG<br>TTTGGGCCATCAGCAATTTTATGGTGTTTGTGTGCATGGCGGGCACTACAATTATT<br>AGCATTGTTTCTATCAACGAATCGTCTGAGGGAGTTCAACACGTCATTGGGGCAA<br>ATAAAGCAACAAAAATTGCCTCACTGGTCGTTTTTGCCCTTCTTGGCATTCCTCTTG<br>CTGTTACGTACAGTGTTCCTTTCTCTGTCACGGCAGAGTTGACGGCTGATGCTGGT<br>GGCGGACAAGGGTTGGCCATAGGAGTTCTCAACCTTGCCATTGTCATACCACAGA<br>TGATTGTATCGCTTGGTGCGGGTCCCTGGGACGCCTTATTTGGTGGAGGGAACAT<br>ACCCGCATTTGCCGTGGCTTCCATTTTTGCCCTCGCTGCCGGTATCATTGCCACTCT<br>CAAGCTGCCAAACCTCGCAAACTCTTATAGGTCATCCGGCTTCCATTTTGGATAA
CrSUT3	AAAATAATGAAACAAACCGACAACAAATCCTCTTTGCAAGTGCAGCAGTCCCCAC<br>CGCCGCCGCAGACACGGTTGTGGAAGGTCATCATGGTAGCGGCCATAGCCGCCG<br>GTATCCAGTTCGGGTGGGCCATCCAACTGTCCCTGCTCACTCCATATGTCCAGCTC<br>CTGGGAATACCTCACAAATACGCACCGCTGATCTGGCTTTGTGGGCCCATCTCCG<br>GTATGTTCGTGCAGCCCATGGTGGGTTATTACAGTGACAACTGCGGCTCCCGCTT<br>CGGTCGCCGTCGTCCATTCATAGCCGCCGGTGCTTCCCTTGTAACCGTAGCTGTCC<br>TGCTTATTGGTTTCGCTGCTGATATCGGACATGTCTCCGGTGATCCTCTCGGGAAA<br>GGGTCTAAGCCTCGGGCCATTGCTGTTTTCGTCGTTGGATTTTGGATCCTCGACGT<br>GGCTAATAACATGTTACAGGGCCCATGCAGAGCACTGCTGGCAGATCTCTCCGGT<br>GGGAAATCGAAGAGGATGCGAACGGCAAACTCTTTCTTCTCCTTCTTCATGGCCG<br>TCGGGAACGTCATGGGTTACGCCGCCGGCTCATACTCCCGGCTTCACAAAATCTT<br>CCCGTTCTCCCAAACGAAAGCATGCGACGTTTACTGTGCAAACTTGAAGAGCTGT<br>TTCTTCATCTCCGCAGCACTGTTACTGTTTTTGACTACCTTGGCCTTGAGCACAGTT<br>CATGAAAGCGAGCTTCCGAAGGACATCGGCGACGCGGCAGAGGACGCCGTTGA<br>CGACGAGAAGCTGTCGTTGCCGGCGGGGAAGTCAAAGCTGCCATTCTTCGGCGA<br>AATATTTGGTGCTCTGAGAGACCTCCCAAGGCCCATGTGGATCCTCCTGCTTGTCA<br>CGTGCCTCAACTGGGTCGCCTGGTTCCCTTTCTTCTTATTTGACACTGACTGGATG<br>GGGAAGGAGGTGTACGGCGGGAATCCCGACGGAAACCCCGCAGAAGGACGGCT<br>GTACGAGGAAGGGGTCCAAACCGGCTCGTTGGGGCTCATGCTCAACTCCGTTGTT

	TTGGGGTTCACGTCGCTGGGAGTGGAGTTGTTGGCTCGGCGGTTCAGCGGCGTC<br>AAGAAGCTGTGGGGCGGCGTCAACTTTCTCTTGGCGGCTTGCTTGGCCATGACAA<br>TTTGGGTTAGCAAAAAGGCGGAGAAATCACGGCGGTACGGAACAGACGGGGCA<br>CTCATGGCGCCGTCGGCGGGAGTCCGTGCCGGAGCATTAACCCTCTTTTCTGTTCT<br>TGGGATTCCTCTCGCGGTGACTTATAGTATTCCCTTCGCTCTGGCATCCATCTTTTG<br>CAGTAACACAGGTGCAGGCCAAGGTCTATCCCTTGGAGTTCTCAATCTTGGAATT<br>GTCATGCCACAGATGATCGTGTCGCTGGTGGGTGGACCATGGGACGAGTTGTTC<br>GGAGGTGGGAACTTGCCTGCATTTATAGTAGGGGCAGTTTCGGCAGCATTGAGT<br>GGGATATTTGCCATCACATTGCTTCCTTCTCCACCACCTGATGTGAAGCCAGGCCT<br>TCCCGTTGCTGCTTTCCACTGAAACGTGTTTGGAAATTACGAATGTCTCCTTTAGTT<br>CATCACCTTTGGGTGTTTCTATTTT

Table S2 Primer sequences used for qRT-PCRs, cloning and probe synthesis for *in situ* hybridization.

**Purpose**	**Primer Name**	**Sequence 5'-3'**
qRT-PCR	CrSUT1 +82 fw	ATAGTGCTGGTGGCTTCCATC
	CrSUT1 +271 rev	ACCAAAATCACCGGTTTCAATGG
	CrSUT2 +1448 fw	GAGTTCAACACGTCATTGGG
	CrSUT2 +1615 rev	TGAGAACTCCTATGGCCAAC
	CrSUT3 +1320 fw	TAACACAGGTGCAGGCCAAG
	CrSUT3 +1557 rev	TCAGTGGAAAGCAGCAACG
	CrSWEET10 +185 fw	TGCCATGCTTTGGATATACTATGC
	CrSWEET10 +365 rev	GTGTGACAAGAACAATAGCACC
	CrActin +497 fw	CCATCTACGAAGGCTATGCC
	CrActin +671 rev	TCGAGAGCAATGTAGGCTAG
	AtSWEET15 +627 fw	TACTAGGGCTGTTGCAAATG
	AtSWEET15 +803 rev	TTGGACAGATCCTCATGATG
	AtUBQ10 +1066 fw	GATGGTCGTACTTTGGCGGATTA
	AtUBQ10 +1130 rev	AGACGCAACACCAAGTGAAGGG
Cloning	CrSUT1 +1 *BsaI* fw	TTGGTCTCACACCATGGAGACAGCCTCTGCTAAC
	CrSUT1 +1515 *BsaI* rev	TTGGTCTCACCTTTCAATGGAAACCTCCGGCC
	CrSUT1 +1512 *BsaI* rev	TTGGTCTCACCTTATGGAAACCTCCGGCCGCAG
	CrSUT2 +1 *BsaI* fw	TTGGTCTCACACCATGATGGATGCGGTGTCGATC
	CrSUT2 +1806 *BsaI* rev	TTGGTCTCACCTTTTATCCAAAATGGAAGCCGG
	CrSUT2 +1803 *BsaI* rev	TTGGTCTCACCTTTCCAAAATGGAAGCCGGATG
	CrSUT3 +1 *BsaI* fw	TTGGTCTCACACCATGAAACAAACCGACAACAAATCCTC
	CrSUT3 +1557 *BsaI* rev	TTGGTCTCACCTTTCAGTGGAAAGCAGCAACGG
	CrSWEET10 +1 *BsaI* fw	TTGGTCTCACACCATGGCTTTCTCTGGTCATAACTG
	CrSWEET10 +888 *BsaI* rev	TTGGTCTCACCTTTCAAGCTTCCACAGCTTGAAG
	CrSWEET10 +885 *BsaI* rev	TTGGTCTCACCTTAGCTTCCACAGCTTGAAGC
	AtSWEET15 -2183 *BsaI* fw	TTGGTCTCACACCTTGCTAGTGAATTATGAATATCTATTGTTCATG
	AtSWEET15 -184 *BsaI* rev	TTGGTCTCACCTTCCATTGTAGTCAAAATATTTTGATTTTAATG
Probes	CrSUT1 +1263 AS fw	TATCTTTTCGAGCAACGCAG

	CrSUT1 +1524 T7 AS rev	GAAATTAATACGACTCACTATAGGGTCACCGGTTTCAATGGAAAC
	CrSUT1 +1263 T7 S fw	GAAATTAATACGACTCACTATAGGGTATCTTTTCGAGCAACGCAG
	CrSUT1 +1524 S rev	TCACCGGTTTCAATGGAAAC
	CrSUT2 +1528 AS fw	GCTGTTACGTACAGTGTTCC
	CrSUT2 +1806 T7 AS fw	GAAATTAATACGACTCACTATAGGGTTATCCAAAATGGAAGCCGG
	CrSUT2 +1528 T7 S fw	AAAGGGAAACCAAGACAACC
	CrSUT2 +1806 S fw	GAAATTAATACGACTCACTATAGGGGCTGTTACGTACAGTGTTCC
	CrSUT3 +515 AS1 fw	CGGCAAACTCTTTCTTCTCC
	CrSUT3 +835 T7 AS1 rev	GTAATACGACTCACTATAGGGGAGCACCAAATATTTCGCCG
	CrSUT3 +277 AS2 fw	CGTCGTCCATTCATAGCCG
	CrSUT3 +515 t7 AS2 rev	GTAATACGACTCACTATAGGGGTTCGCATCCTCTTCGATTTC
	CrSUT3 +1119 AS3 fw	CTTTCTCTTGGCGGCTTGC
	CrSUT3 +1367 T7 AS3 rev	GTAATACGACTCACTATAGGGCCAAGATTGAGAACTCCAAGGG
	CrSUT3 +515 T7 S fw	GTAATACGACTCACTATAGGGCGGCAAACTCTTTCTTCTCC
	CrSUT3 +835 S rev	GAGCACCAAATATTTCGCCG
	CrSWEET10 +1 AS1 fw	ATGGCTTTCTCTGGTCATAACTG
	CrSWEET10 +215 T7 AS1 rev	GAAATTAATACGACTCACTATAGGGAGAAGAGTGGTGTTTGAGGTTTTG
	CrSWEET10 +358 AS2 fw	AGGGTTTGGTGCTATTGTTCTTG
	CrSWEET10 +548 T7 AS2 rev	GAAATTAATACGACTCACTATAGGGTAGAAGAACCACATCACAGCAC
	CrSWEET10 +642 AS3 fw	GTTGGTCTCTACATGGTGTACAAG
	CrSWEET10 +827 T7 AS3 rev	GAAATTAATACGACTCACTATAGGGATTTTGTCCGAACACACGAGTG
	CrSWEET10 +1 T7 S fw	GAAATTAATACGACTCACTATAGGGATGGCTTTCTCTGGTCATAACTG
	CrSWEET10 +215 S rev	AGAAGAGTGGTGTTTGAGGTTTTG

References

Le, S. Q., & Gascuel, O. (2008). An improved general amino acid replacement matrix. *Mol Biol Evol*, *25*(7), 1307-1320. https://doi.org/10.1093/molbev/msn067

Tamura, K., Stecher, G., & Kumar, S. (2021). MEGA11: Molecular Evolutionary Genetics Analysis Version 11. *Mol Biol Evol*, *38*(7), 3022-3027. https://doi.org/10.1093/molbev/msab120
